# Hybrid Synthetic Minority Over-sampling Technique (HSMOTE) and Ensemble Deep Dynamic Classifier Model (EDDCM) for big data analytics

**DOI:** 10.1038/s41598-025-23062-3

**Published:** 2025-11-11

**Authors:** Priyadharsini M, Bhawana Tyagi, Naga Priyadarsini R, Mohankumar B

**Affiliations:** School of Computer Science and Engineering, Vellore Institute of Technology, Vellore, Tamilnadu India

**Keywords:** Feature selection, Optimization Ensemble Feature Selection Model, Fuzzy Weight Dragonfly Algorithm, Adaptive Elephant Herding Optimization, Fuzzy Weight Grey Wolf Optimization, Ensemble Deep Dynamic Classifier Model, Engineering, Mathematics and computing

## Abstract

Big Data Classification (BDC) has become increasingly important across domains such as healthcare, e-commerce, and banking. However, challenges such as high dimensionality and class imbalance often degrade the performance of conventional machine learning (ML) models. This study proposes a hybrid framework that integrates meta-heuristic optimization with class imbalance handling to enhance BDC effectiveness. To address the class imbalance problem in both binary and multi-class datasets, a Hybrid Synthetic Minority Over-sampling Technique (HSMOTE) is introduced. HSMOTE generates synthetic minority samples by interpolating between closely located minority instances, improving the representation of rare classes. For robust feature selection, the Optimization Ensemble Feature Selection Model (OEFSM) is developed by combining the outputs of three algorithms: Fuzzy Weight Dragonfly Algorithm (FWDFA), Adaptive Elephant Herding Optimization (AEHO), and Fuzzy Weight Grey Wolf Optimization (FWGWO). These algorithms contribute diverse search strategies to improve feature relevance and reduce redundancy. To handle classification, the Ensemble Deep Dynamic Classifier Model (EDDCM) is proposed. EDDCM incorporates three deep learning (DL) architectures Density Weighted Convolutional Neural Network (DWCNN), Density Weighted Bi-Directional Long Short-Term Memory (DWBi-LSTM), and Weighted Autoencoder (WAE). Their outputs are aggregated using a dynamic ensemble strategy that considers both accuracy and diversity to improve final prediction reliability. All models are implemented in MATLAB (2014a), and performance is evaluated using precision, recall, F-measure, and accuracy. The proposed framework demonstrates improved classification results across various datasets, particularly under conditions of imbalance and high dimensionality.

## Introduction

For the majority of the Data Mining (DM) and ML algorithms in use today, learning from very large databases is a significant challenge. The phrase “BD” describes the challenges and drawbacks of processing and analyzing vast volumes of data, and is frequently used to describe this issue. The vast amounts of original information saved have garnered significant interest across various fields, including bioinformatics, health, marketing, and finance. Because of its capacity to extract unexpected information, artificial intelligence (AI) approaches have recently attracted a lot of attention in a variety of applications^1^.

Deep learning (DL) methods have lately been effectively utilised in Breast Cancer (BC) Detection since the development of AI. It increases the likelihood of a patient’s survival by facilitating early diagnosis. For comparable Feature Extraction (FE), DL necessitates less human involvement than traditional ML methods^2^.

FS algorithms are assessed using a new frequency-based stability metric known as Rank stability (RS). Both the feature ranking and subsets of features are considered. Following a little alteration to the training set, the suggested metric assesses the variation in feature ranking produced by FS algorithms. Used real-world datasets to investigate different heterogeneous ensemble approaches and compare them with conventional FS methods^3^. ML methods are frequently utilized to learn, forecast, and categorize data in this environment^4^. BD provides the academic community with opportunities to find novel concepts^5^. This study discusses the applications of ML classifiers in many application domains, which include cybersecurity and healthcare^6^. Several features (referred to as attributes in dataset nomenclature) make up real-world problems in a variety of fields. Not all of these variables are crucial, though, as some are unnecessary or redundant, which might impact ML classifier performance^7^. To improve ML performance, FS is a feature selection method for selecting the relevant features that can decrease the dimension of data^8^. The FS process is initiated using a search approach to find feature subsets. Several performance metrics, such as classification accuracy, are then used to evaluate feature subsets. A terminating criterion, such as the maximum number of generations, terminates the feature selection procedure. The accuracy of the chosen subset of features can be evaluated using a validation technique at the final stage of the feature selection procedure. Search techniques and subset performance evaluations constitute the foundation of FS methods. Using a tool to choose subsets of the search strategy’s features is the first stage. The selection of features must achieve two goals: maximising output accuracy performance and minimising or eliminating the number of selected features.

Individual assessments or the evaluation of subsets can serve as the foundation for the analysis of features from datasets^9^. While alternative methods use search tactics to produce a number of feature subsets, individual assessments evaluate features according to their significance. Until a final selection of chosen attributes is reached, these subsets are evaluated iteratively using optimality criteria^10^.

BD Analytics (BDA), heavily relies on ML methods and processing capacity. In order to predict future data, ML learnt patterns and generalised them, focussing on input data representation^11^. The performance of ML is significantly impacted by the data representation. A simple machine learner may still perform well if the data is represented well, but a sophisticated, complex machine learner may perform worse if the data is poorly represented. Using ML techniques, DL automatically uncovers patterns and structures that are hidden in the raw data. Because of its features, DL has gained industry recognition in addition to drawing scholars from various fields. While ML has been effective at some rates in several areas, DL has had far better performance^12,13^. Because it can yield quick and effective results, it is especially popular in the classification of big datasets. To determine which attribute set is best suited for the decision-making task, a pre-training and fine-tuning procedure is employed^14^. DL models: A reliable DL model for low-quality information, incremental DL models for Real-Time information, large-scale DL models for BD, multi-modal DL models, and deep computation models for heterogeneous information are the DL models that have frequently been used for BD feature analysis^15,16^.

Hybrid Synthetic Minority Over-Sampling Technique (HSMOTE) and an Ensemble Deep Dynamic Classifier Model (EDDCM) together address a core bottleneck in big data analytics: severe, evolving class imbalance under high-velocity, high-variety streams. Real-world platforms—fraud detection, intrusion monitoring, medical triage, and IoT telemetry produce skewed distributions where rare but critical events are easily drowned out. Conventional SMOTE variants help, yet often inject noisy or redundant samples and fail to adapt when the data distribution drifts. HSMOTE remedies this by combining density-aware synthesis with selective cleaning (e.g., Tomek/ENN-style filtering) to preserve minority manifolds while pruning borderline and overlapping regions. Unlike static resampling, HSMOTE is designed for mini-batch and streaming regimes, recalibrating synthesis ratios as drift and rarity patterns change. On the modeling side, EDDCM employs a pool of heterogeneous deep learners lightweight CNN/MLP blocks, GRU/LSTM for sequences, and attention layers for long-range dependencies. A dynamic orchestration layer updates base-learner weights online using recent validation windows, uncertainty cues, and cost-sensitive risk. This design emphasizes recall and AUC-PR without sacrificing calibration, enabling actionable thresholds in operational settings. To curb overfitting to synthetic regions, EDDCM integrates manifold mixup and focal/cost-sensitive losses aligned with HSMOTE’s locality structure. Model snapshots are periodically distilled into compact students, reducing latency for edge and near-real-time deployment. The pipeline scales on Apache Spark/Ray, leveraging GPU acceleration and asynchronous data loaders for throughput. Feature drift and covariate shift are tracked via population stability/energy distances, triggering resampling and ensemble refresh policies. Robustness is improved through adversarial augmentation around minority boundaries to harden decision frontiers. Explainability is supported with SHAP/IG summaries computed on minority instances to expose drivers of rare-event predictions. Fairness checks ensure that synthetic sampling does not amplify group biases, with constraints baked into the resampling schedule. Comprehensive evaluation targets heavily imbalanced benchmarks and real logs, reporting AUC-PR, G-mean, F1-minority, ECE, and tail-latency. Ablations isolate gains from hybrid cleaning, density-aware synthesis, and dynamic ensembling under controlled drift scenarios. Operationally, streaming checkpoints and rollback safeguards enable safe updates in live pipelines. By uniting HSMOTE’s data-space corrections with EDDCM’s adaptive model-space learning, the framework yields stable rare-event detection at scale. This paper details the design, algorithms, and empirical benefits of the HSMOTE-EDDCM stack for modern big data environments. In real-world applications such as healthcare, e-commerce, and finance, the effectiveness of classical ML algorithms is hindered by the difficulty of class imbalance in BDC. When faced with unbalanced datasets, standard approaches like K-nearest Neighbors (KNN), Support Vector Machine (SVM), and Synthetic Minority Oversampling Technique (SMOTE) often provide incorrect classifications. When dealing with high-dimensional data, classic feature selection approaches also fail, and ensemble methods can’t reach their full potential since they don’t have dynamic integration. Finding a better framework to optimize feature selection and classification performance while dealing with unbalanced datasets is the main issue this study aims to solve. In addressing these difficulties, our study makes several significant contributions. Firstly, this research addresses the shortcomings of classic SMOTE by introducing the HSMOTE, which generates more realistic synthetic minority samples and hence improves class balance. Also, to improve the model’s accuracy and computational efficiency, an OEFSM is suggested. This model employs sophisticated metaheuristic algorithms to choose the most important features. Third, to improve the model’s generalizability and resilience, the EDDCM is created by merging several DL classifiers with dynamic voting. Finally, comprehensive studies on benchmark datasets confirm the efficacy of the suggested strategy, showing that it outperforms state-of-the-art approaches in terms of accuracy, precision, recall, and F-measure.

## Literature review

An ensemble for FS based on aggregating feature rankings in an attempt to solve the challenge of choosing the best ranker approach for each problem^17^. The ensemble’s adequacy was then evaluated using SVM as the classifier after the results of the individual rankings were aggregated with SVM Rank. The suggested ensemble outperforms or performs comparably to the FS techniques used separately, according to results on five UCI datasets.In this work, based on agreements using OBEFS, some relevant features are chosen. Results from several FS techniques, such as FMBOAs, LFCSAs (Levy Flight search algorithms), and AFAs (Adaptive Firefly algorithms), can be combined using ensembles. Through the selection of optimised feature subsets, these methods generate 3 feature subsets, which are then matched for correlations. For classifications, OBEFS trained on FCBi-LSTMs (fuzzy convolution Bi-LSTM) produce the best features.

### Literature review on Matthew correlation coefficients

F-measure values, MCC (Matthew correlation coefficients), and (Leave-One-Person-Out) LOPO—CV (Cross Validations) are used to assess the methodologies used in this work’s suggested model, thereby facilitating use of the University of California-Irvine (UCI) learning repository. In addition to a structured taxonomy that classifies current approaches according to essence, exploration approach, implementation procedure, and attribute model, In^18^ authors offer a thorough analysis of the most recent FS approaches in the context of BD. According to their learning task, motivation, framework, exploration approach, model, robustness, and shortcomings, it offers a qualitative evaluation of FS techniques. To demonstrate the quantity of publications about FS according to the primary category, timeframe, and other subcategories, a quantitative analysis is conducted subsequently. Using 12 benchmark datasets from the UCI ML Repository and Arizona State University (ASU) FS Repository, an experimental study is also carried out to compare ten methods from various categories. By the Acc, P, R, F-score, also the amount of chosen attributes, performance was assessed. To choose useful features from an input dataset, a thorough and effective univariate EFS (uEFS) methodology was suggested by Ali et al.^19^. After a thorough assessment of a feature set, the unified features scoring (UFS) algorithm is suggested as the way for producing a last classified feature set for the uEFS methodology. A threshold (T) value selection (TVS) technique is given in selecting a subset of features for which it is perceived important to design the classifier that would define the cutoff points, eliminating irrelevant features. The standard benchmark datasets are used to assess the uEFS approach. In comparison to state-of-the-art (SOTA) methodologies, outcomes of experiments demonstrate that the suggested uEFS methodology delivers competitive Acc and achieves (1) an average rise of approximately 7.00% in f-measure and (2) an average increase of approximately 5.00% in predictive Acc.

### Literature review on eXtreme Gradient Boosting

A Sort Aggregation-based EFS (SA-EFS) method focused on classification challenges was introduced by Wang et al.^20^. The outcomes of three FS techniques, specifically the Chi-Square Test (CST), the maximum data coefficient, and eXtreme Gradient Boosting (XGBoost), are combined according to a particular approach for High-Dimensional (HD) data sets. The integration effects on this framework of arithmetic mean (AM) and geometric mean (GM) aggregation procedures are studied. Three classifiers, KNN, RF (Random Forest), and XGBoost, each with excellent performance, are used to test the classification and detection capability of the feature subset. For different classifiers, the impact of T on the classification performance is tested. Numerical outcome show that the AM aggregation EFS can greatly improve the classification accuracy compared to the single FS method. It is recommended to use this T interval value of 0.1.

To improve the classification, Chandralekha and Shebagavadivu^21^ suggested an EFS method that uses wrapper techniques and Random Trees (RT). The suggested EL classification approach uses RT, bagging, and the wrapper method to generate a subset. For choosing the optimal attributes over classification, the suggested approach employs probability weighting criteria and removes features that aren’t relevant. The enhanced algorithm can enhance classification performance by differentiating between relevant and irrelevant features. The suggested approach outperforms the other ensemble approaches and reaches a mean classification accuracy of 92%.

### Literature review on Neural Network

Elgin Christo et al.^22^ have developed a correlation-based EFS technique to choose the best features from the three feature subsets. The selected top features using correlation-based EFS are used to train a Gradient Descent Backpropagation (NN) Neural Network (GD-BPNN). The ten-fold CV technique has been used for training and evaluating the classifier’s performance. The accuracy of the classifier has been assessed using the Wisconsin Diagnostic BC (WDBC) dataset and the Hepatitis dataset from the UCI ML repository. To assist clinicians in making clinical diagnoses, the suggested architecture can be modified to develop clinical DM systems for each disease.

A two-step combination technique for Gene Expression (GE) in a range of disorders has been suggested by Rezaee et al.^23^: A novel Deep NN (DNN) is used to classify the genes, and soft ensembling is applied for finding the most efficient genes. The proposed FS method builds a highly generalizable model along with low error levels through the combination of three different ways of choosing and ranking wrapper genes based on the kNN algorithm. In three microarray datasets of prostate cancer, leukaemia, and diffuse large cell lymphoma, the most efficient gene subsets were found using soft ensembling. All three datasets used a stacked DNN. To further evidence the generalisability of the model method, two previously unseen datasets were analyzed: one of multiple sclerosis-related brain tissue lesions and the other of small, round blue cell tumors (SRBCT).

### Litrature review on machine learning

Rashid et al.^24^ introduced a new Random Feature grouping (RFG) with three variants to dynamically decompose BD datasets and guarantee the likelihood of grouping interacting features into the same subcomponent. Cooperative Co-Evolutionary-Based FS with RFG (CCFSRFG) is its name since it can be used in CC-based FS processes. Six well-known ML classifiers were tested using seven distinct datasets, some with and some without FS, from the Princeton University Genomics library and the UCI ML repository. In the majority of cases, as shown by the experimental results, they include kNN, J48, RF, SVM, and naïve Bayes (NB). When compared to the current solution, CCEAFS, a CC-based FS that takes into account all qualities, the suggested system performs better. For ensemble learning, you and colleagues^25^ introduced a two-stage weighted EL approach that uses the PSO algorithm to strike a balance between diversity and accuracy. By adjusting datasets and input features with a mixed-binary PSO algorithm, the primary objective of the first stage is to optimize individual learner diversity. The second step involves using a weighted ensemble method to enhance the accuracy of the ensemble classifier further. This method strikes a balance between diversity and accuracy. Experimental results on 30 UCI datasets show that the suggested approach outperforms other SOTA established standards, and the optimization of the weighted ensemble set of classifiers is done using the Particle Swarm Optimization (PSO) algorithm.

### Litrature review on cardiovascular disease

A multi-layer dynamic system (MLDS) that may improve its knowledge in each layer was suggested by Uddin and Halder^26^. The recommended FS framework makes use of the following tools: Correlation Attribute Evaluator (CAE), Gain Ratio Attribute Evaluator (GRAE), Information Gain Attribute Evaluator (IGAE), Lasso, and Extra Trees classifier (ETC). Combining RF, NB, and Gradient Boosting (GB) creates the model’s ensemble technique for classification. Although none of the previously described base classifiers were successful in any layer of classification, the KNN method was employed to locate data points in the test data’s immediate vicinity. In addition to the Long Beach dataset, the model has been trained using the Cleveland dataset and the Hungarian dataset. The accuracy of the model improved with different dataset splitting percentages. The proposed model outperformed five competing models in predicting the occurrence of cardiovascular disease (CVD).

For unbalanced data classification problems, Wang et al.^27^ proposed a novel Improved Deep-Ensemble-level-based Takagi–Sugeno–Kang (TSK) fuzzy classifier (IDE-TSK-FC) that stacks Zero-Order TSK fuzzy subclassifiers on the minority class. The original training dataset, IDE-TSK-FC, is the basis for the first interpretable ZO TSK fuzzy subclassifier. Finally, a sequence of additional ZO TSK fuzzy subclassifiers is layered layer-wise using the newly detected issues from the training dataset and related interpretable predictions generated through averaging on all previous layers^28–30^. A real-world health care dataset and public datasets^31,32^were used to demonstrate the effectiveness of IDE-TSK-FC in class-imbalanced learning. Classifier performance is measured against that of ZO TSK fuzzy classifiers as shown In the Table 1.

**Table 1:** Comparision analysis of literature survey.

Study/method	Key issue in previous methods	Proposed solution/approach	How the issue was resolved/alleviated
Wang et al.^20^—SA-EFS (Sort Aggregation-based EFS)	Single FS methods struggle with stability and accuracy in HD datasets	Combined CST, Maximum Data Coefficient, and XGBoost via AM & GM aggregation	AM aggregation improved accuracy significantly compared to single FS, with optimal T interval (0.1) enhancing robustness across classifiers
Chandralekha and Shebagavadivu^21^—Wrapper + Random Trees EFS	Traditional FS often selects irrelevant features, reducing classifier accuracy	Wrapper-based RT + bagging + probability weighting to refine feature selection	Removed irrelevant features, achieving better attribute selection and mean classification accuracy of 92%, outperforming other ensemble methods
Elgin Christo et al.^22^—Correlation-based EFS + GD-BPNN	Existing FS methods fail to consider correlation and domain-specific datasets	Correlation-based EFS + Neural Network (GD-BPNN) with tenfold CV	Improved disease classification on WDBC & Hepatitis datasets; adaptable for clinical DM systems, addressing feature redundancy issues
Rezaee et al.^23^—Two-Step Gene Expression FS + DNN	Gene expression FS suffers from poor generalizability and high error rates	Wrapper-based gene ranking (kNN) + soft ensembling + stacked DNN	Found efficient gene subsets, reduced error rates, and validated generalizability on unseen MS and SRBCT datasets
Rashid et al.^24^—Random Feature Grouping (RFG) + CCFS	Existing CC-based FS ignores feature interactions, reducing accuracy	Introduced RFG variants within CCFS to dynamically group interacting features	Improved accuracy across 7 datasets with kNN, J48, RF, SVM, NB, outperforming baseline CC-based FS (CCEAFS)
You et al.^25^—PSO-based Two-Stage Weighted Ensemble	Difficulty balancing diversity vs. accuracy in ensemble classifiers	Stage 1: Mixed-binary PSO for learner diversity. Stage 2: Weighted ensemble optimization	Struck balance between diversity & accuracy; outperformed SOTA methods on 30 UCI datasets
Uddin and Halder^26^—Multi-Layer Dynamic System (MLDS)	Base classifiers underperform in CVD prediction due to weak FS	Multi-layer FS (CAE, GRAE, IGAE, Lasso, ETC) + Ensemble (RF, NB, GB) + KNN for local refinement	Improved predictive accuracy on Cleveland, Hungarian & Long Beach datasets; surpassed 5 baseline models
Wang et al.^27^—IDE-TSK-FC (Improved Deep-Ensemble TSK Fuzzy Classifier)	Class-imbalanced data weakens classifier learning, esp. minority classes	Layered Zero-Order TSK fuzzy subclassifiers with ensemble stacking	Enhanced minority-class detection; real-world & public datasets showed better performance vs. standard ZO TSK classifiers

Moradi et al.^33^ presented a new PSO-based Hybrid FS that combines PSO with a Local Search method, the HPSO-LS technique. With its correlation data, the HPSOLS strategy selects the less-correlated yet more important feature subset, and the LS method guides the PSO search to identify unique features. Additionally, the suggested method employs a subset size determination process to choose a smaller subset of features^34,35^. To evaluate the efficacy of the suggested method, we compare it to five SOTA FS approaches and thirteen benchmark classification issues^36^. In addition, HPSO-LS has been tested against five popular wrapper-based methods: GA, PSO, Simulated Annealing (SA), and Ant Colony Optimisation (ACO), as well as four popular filter-based methods: Information Gain (IG), term variance, Fisher Score (FS), and mRMR.

The obtained results demonstrated that the suggested method achieves higher classification accuracy than the filter-based and wrapper-based FS approaches. Results from multiple statistical tests also show that the suggested strategy is significantly better than alternatives. For a classification task, Shukla et al.^37^ proposed a new hybrid FS technique named Filter-Wrapper FS (FWFS). It also describes the limitations of current approaches^38,39^. In the proposed model, it selects the high-ranked feature subset based on the front-end filter ranking technique that is known as Conditional Mutual Information Maximisation (CMIM)^40,41^. However, an effective method called Binary GA (BGA)^42,43^ has accelerated the search in identifying significant feature subsets. The suggested approach has the merit of applying a learning model over the chosen subsets of features, while an exhaustive strategy, on its part, speeds up the process of FS^44–46^ without losing much classification accuracy on a much smaller dataset^47–49^.

### Literature review on Whale Optimization Algorithm

Pradip Dhal and Chandrashekhar Azad^50^ suggested a multi-objective evolutionary feature selection approach, Whale Optimization Algorithm (WOA), for the classification of multi-label data. The author has used the tournament search to choose a new whale instead of WOA’s random search. The process of Tournament Selection (TS) comprises distributing a subset of the population to several “tournaments” chosen at random. Maximizing the Jaccard similarity and minimizing the specified characteristics are the two goals of the multi-objective criteria in this case. The author has used multi-label datasets from several domains to verify the method’s resilience. To round up the research, the author compared the suggested approach to many classic ML and multi-label classifiers. The suggested FS provides competitive performance, particularly when labels are constrained, according to empirical findings on popular multi-label datasets.

### Literature review on CNN

Pradip Dhal and Chandrashekhar Azad^51^ proposed a multi-stage multi-objective GWO based feature selection approach for multi-label text classification. First of all, FS is not stable with the current sample size and HD. Additionally, FS decelerates with HD. The third consideration is that the Classification Accuracy (CA) that a given FS method produces can be inadequate. A Meta-heuristics Algorithm (MA) for MTC based on a two-stage FS method is presented in this work. Both the first and second stages are based on the multi-objective GWO algorithm; the first stage is FS-based, and the second stage is filter-based. One goal is to reduce the Selected Features (SF), while the other is to reduce the Hamming Loss (HL). For this categorization job, the author has used the Multi-Layer Perceptron (MLP) model. The results of the experiments show that the proposed FS technique provides better HL while using fewer features.

Pradip Dhal et al.^52^ recommended the deep ensemble-based framework for the prediction of oral cancer through histopathological images. For deep feature extraction, the suggested approach uses several DL methods, including CNNs, Bi-LSTM, and Bidirectional Gated Recurrent Units (Bi-GRUs). For this purpose, we have built CNN and Bi-LSTM blocks to analyze histopathology pictures and extract contextual and spatial characteristics. The suggested Bi-GRU block, when combined with CNNs, improves classification performance by making use of sequential and spatial characteristics to more accurately represent visual dependencies. Combining the deep features obtained from the suggested CNN, Bi-LSTM, and Bi-GRU blocks produces an additional set of deep features. Impressive accuracy scores of 98.34% for the HOCDD dataset and 97.89%, 98.76% for the HRNEOCD Set-1, Set-2 datasets were achieved by the proposed ensemble-based classification model by making good use of deep features. This demonstrates the model’s strong predictive capabilities and its potential for reliable OC prediction.

Pradip Dhal and Chandrashekhar Azad^53^ discussed the Zone Oriented Binary Multi-Objective Charged System Search Based Feature Selection Approach for Multi-Label Classification. A hybrid multi-objective FS technique based on CSS and GWO methodologies is proposed in this study for the MLC issue. Two goals have been defined: reducing the number of features in the feature set and minimizing the Hamming loss (HLoss) value. Along with this, we have introduced a new feature zone that categorizes features into informative and non-informative types. To the goal function of the FS approach, we have integrated the Preference Ranking Organization METHOd for Enrichment of Evaluations (PROMETHEE) method. For the modified charge particles in the CSS algorithm, we have included the new velocity equation here. A revised velocity equation that incorporates the GWO attribute enhances the CSS algorithm’s exploration and exploitation capabilities. We have used six publicly available multi-label datasets for experimental verification: CAL500, Emotions, Medical, Enron, Scene, and Yeast. In terms of several performance indicators, the results demonstrate that the suggested method yields the best value. For CAL500, the suggested technique obtains an ideal Jaccard Score (JC) and HLoss value of 0.4408 and 0.0645; for Emotions, 0.84169 and 0.0719; for Medical, 0.9486 and 0.0019; for Enron, 0.5950 and 0.0205; for Scene, 0.7391 and 0.0495; and for Yeast, 0.6452 and 0.0766.

Pradip Dhal et al.^54^ introduced a clinical diabetes prediction support system based on a multi-objective metaheuristic-inspired fine-tuning deep network. MOODM-Net, a deep network for DM prediction based on Multi-Objective Optimization (MOO), is introduced in this study. The most useful features from various data sources are identified using a unique hybrid Feature Selection (FS) technique that combines multi-objective Harris Hawk Optimization (HHO) with GWO. Data fusion in smart healthcare relies on this FS phase, which seeks to derive useful insights from disparate sources of information, including genetic data, wearable sensors, and electronic health records (EHRs). The suggested deep network is fine-tuned using a hybrid exploration–exploitation technique to maximize its performance for accurate DM prediction using these meticulously chosen characteristics. Validation experiments on two popular DM datasets show that MOODM-Net achieves better prediction accuracy than previous methods.

The NB classifier, which functions as a fitness function (FF), evaluates the effectiveness of the suggested (FWFS) approach. On five biological datasets and five UCI datasets with varying dimensionality and number of occurrences, the efficacy of the chosen feature subset is assessed using multiple classifiers. The experimental findings highlight how the suggested approach outperforms the current approaches and offers more evidence for the notable feature reduction.

Several problems with current BDC methods, especially when handling unbalanced datasets, are what prompted this study. Due to their bias toward the majority class, traditional classification algorithms like KNN and SVM struggle to address class imbalance issues^55^. Although SMOTE and similar approaches try to address this problem, they often provide inaccurate synthetic minority samples that lack diversity, leading to poor model performance^56^. In addition, feature selection algorithms often have issues with scalability, accuracy, and flexibility. This is particularly true in high-dimensional datasets, where the model might be overwhelmed with irrelevant characteristics^57^. Traditional ensemble approaches also tend to misuse or underutilize weak classifiers, which results in inefficiencies, even if they are popular for performance improvement. Since most ensemble systems do not utilize dynamic classifier weighting, the model’s overall performance is compromised because stronger classifiers are not afforded sufficient influence^58^. In addition, many of these approaches don’t utilize DL models, which excel at uncovering hidden connections and patterns in data. To tackle these problems, our method incorporates multiple classifiers with dynamic weighting into an EDDCM, an OEFSM to make feature selection more stable, and a HSMOTE to handle class imbalance better^59,60^. As a feature selection technique, nature-inspired meta-heuristic algorithms emerged as a popular method for selecting optimal features and improving classification performance due to their high robustness and efficiency in exploiting and exploring the vast feature space^61,62^.

As a result of this integration, we can better manage high-dimensional, unbalanced datasets than our present approaches, and our generalization and accuracy will be much enhanced. Our goal is to fill these gaps and provide a BDC solution that is more powerful, efficient, and scalable.

## Proposed methodology

**Fig. 1:**
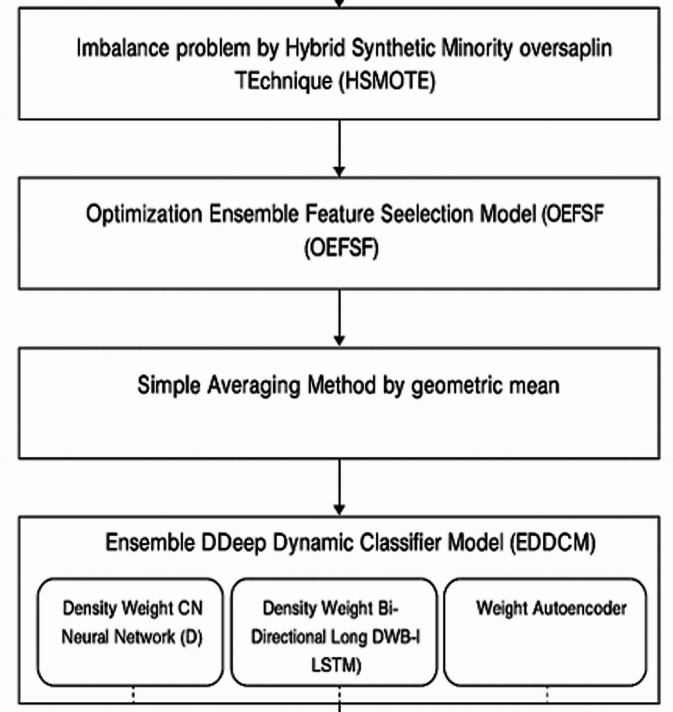
Flow process of the proposed framework.

The Hybrid Synthetic Minority Over-sampling Technique (HSMOTE) has been introduced for solving class imbalance problems. Between minority instances that are near to one another, this kind of oversampling creates new artificial instances. Then, the Optimization Ensemble Feature Selection Model (OEFSM) and Ensemble Deep Dynamic Classifier Model (EDDCM) are introduced for FS and feature classification. The OEFSM system is proposed by combining three different selection algorithms, such as Fuzzy Weight Dragonfly Algorithm (FWDFA), Adaptive Elephant Herding Optimization (AEHO), and Fuzzy Weight Grey Wolf Optimization (FWGWO). An EDDCM system can be created using several different classification methods, such as Density Weight CNN (DWCNN), Weight Autoencoder (WAE), and DWBi-LSTM. Metrics like calculation time, P, R, F-measure, and classification accuracy were used to analyse the results (see Fig. 1).

### Hybrid sampling (HS) for data pre-processing

**Fig. 2:**
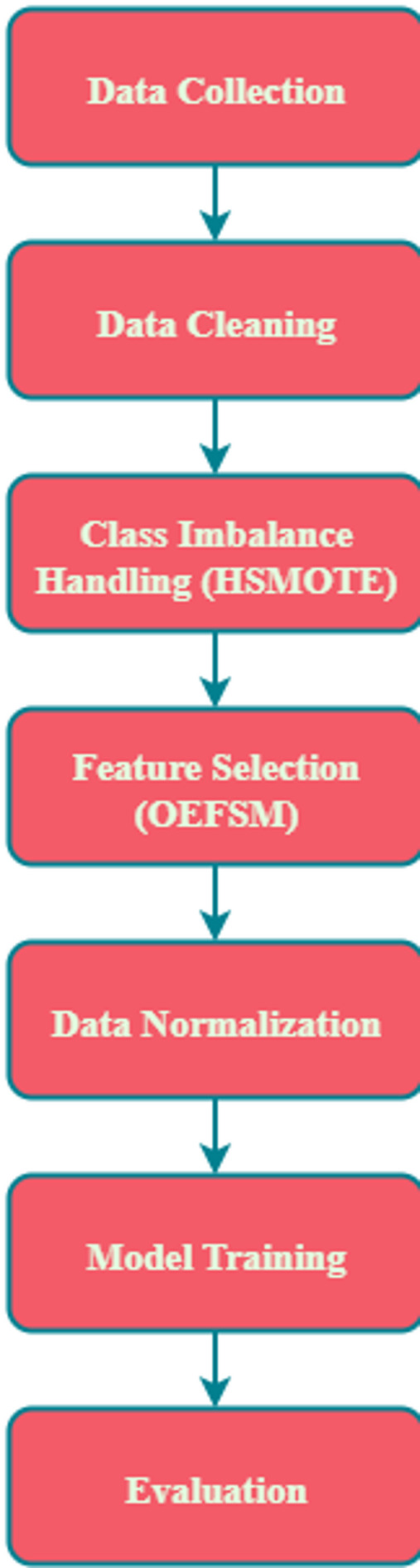
Data pre-processing steps.

HS algorithm combining Misclassification SMOTE (MSMOTE) and kNN based on EDDCM. Depending on the change in the classification metric, EDDCM is employed for classifying samples following HS, regardless of whether it stops at the sampling iteration. Figure 2 shows the data pre-processing steps.

#### Over-sampling (OS)—MSMOTE

One well-known OS algorithm is SMOTE^28^. The kNN algorithm serves as its foundation. Following Eq. (1), a new minority class sample is created by selecting sample ${\text{S}}_{{{\text{j}}\_{\text{min}}}}$ from the kNN of each minority class sample ${\text{S}}_{{{\text{i}}\_{\text{min}}}}$.

1 $${\text{S}}_{{{\text{new}}}} = {\text{S}}_{{{\text{i}}\_{\text{min}}}} + {\text{rand}}\left( {0,1} \right)\left( {{\text{S}}_{{{\text{j}}\_{\text{min}}}} + {\text{S}}_{{{\text{i}}\_{\text{min}}}} } \right),{\text{ i}} = 1, \ldots {\text{n}},{\text{ j}} = 1, \ldots {\text{k}}$$

where rand is random number with iterations (0,1) is represented by rand (·). In general, k = 5 and a newly synthesised sample is represented by S_new_. Based on the samples’ imbalance rate, SMOTE determines how many samples in the ${\text{min}}$ class must be synthesised. The OS rate in SMOTE is determined by the samples’ imbalance rate^29^. As an alternative to using the imbalance rate as the OS rate, the suggested method uses the misclassification (Mmis-class) rate of samples.

During the interval selection procedure, the OS ratio is strongly adjusted via the Mmis-class of RF rather than the unbalance level of the models.

The two sample classes’ M_mis-class_ rates are computed. The amount of M_mis-class_ of a particular sample type is represented by M_mis-class_, which is computed using Eq. (2).

2 $${\text{M}}_{{{\text{mis}}\_{\text{class}}}} = {\text{M}}\left( {{\text{S}}_{{{\text{class}}}} } \right) = \mathop \sum \limits_{{{\text{i}} = 1}}^{{\text{n}}} \left( {{\text{Class}}\left( {{\text{S}}_{{\text{i}}} } \right) \ne {\text{C}}\left( {{\text{S}}_{{\text{i}}} } \right)} \right){ }$$

Here, the original samples’ class is denoted by Class (S_i_). The class of samples that RF has identified is C (S_i_). Equation (3) allows for the following calculation of the RF M_mis-class_ rate for training samples:

3 $${\text{M}}_{{{\text{mis}}\_{\text{rate}}}} = \frac{{{\text{M}}_{{{\text{mis}}\_{\text{maj}}}} }}{{{\text{M}}_{{{\text{mis}}\_{\text{min}}}} }} \times 100{\text{\% }}$$

Here, the amount of ${\text{maj}}$ samples that were misclassified is ${\text{M}}_{{{\text{mis}}\_{\text{maj}}}}$, and the amount of ${\text{min}}$ samples that were misclassified is ${\text{M}}_{{{\text{mis}}\_{\text{min}}}}$. Equation (4) indicates that MSMOTE views the rate of sample M_mis-class_ as the OS rate, which fully utilises the inherent features of samples to increase the recognition of the synthesised ${\text{min}}$ samples. The following are the steps in MSMOTE as shown in the algorithm 1.

:

**ALGORITHM 1:**
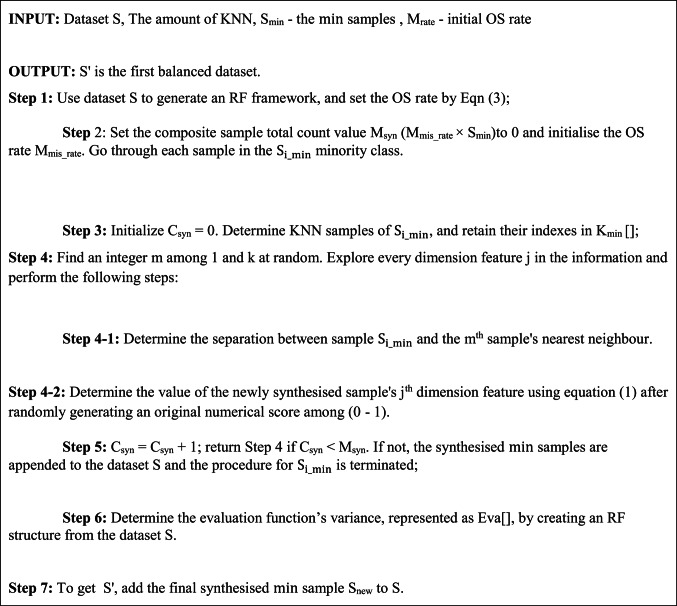
MSMOTE ALGORITHM

#### Under-sampling (US)—kNN

By lowering the quantity of ${\text{maj}}$ samples, the classification efficiency of ${\text{min}}$ samples was enhanced by the US algorithm. According to the nearest neighbor rule, every ${\text{maj}}$ sample’s NN samples are located based on the distance between two samples, and the consistency of their labels is used to find if the ${\text{maj}}$ samples are noise samples. The distance between the kNN sample of S_i_ and S_j_ is greater than the distance between the kNN sample of Si and the sum of the samples in dataset S. Equation (4) represents it.

4 $${\text{kNN}}\left( {{\text{S}}_{{\text{i}}} ,{\text{k}}} \right) = \{ {\text{y}}_{{\text{i}}} \in {\text{S}}|{\text{dist}}\left( {{\text{S}}_{{\text{j}}} ,{\text{S}}_{{\text{i}}} } \right) \le {\text{dist}}\left( {{\text{S}}_{{\text{i}}}^{\prime} ,{\text{S}}_{{\text{i}}} } \right)$$

Here, the distance among sample S_i_ and their neighbour, typically the (ED) Euclidean Distance, is denoted by ${\text{dist}}$, and ${\text{S}}_{{\text{i}}}^{\prime}$ is kNN sample of S_i_ in S. Eliminating majority of the noise samples is the algorithm’s main goal. The following are the steps in kNN as shown in the algorithm 2.

:

**ALGORITHM 2:**
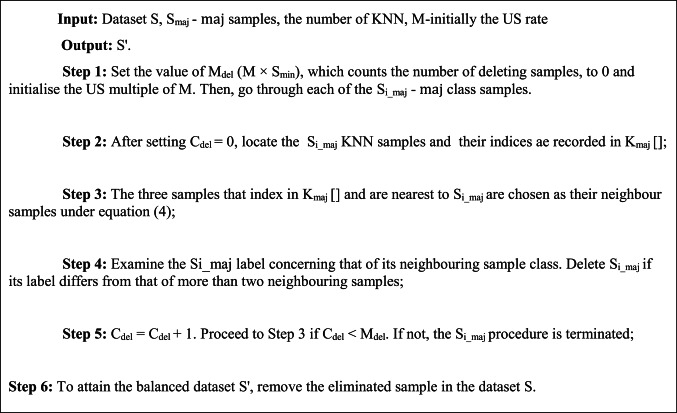
kNN ALGORITHM

#### HS algorithm

This study proposes the MSMOTE-kNN HS algorithm. The accuracy metric was utilized as the interval terminating condition in the procedure to fully account for the samples’ classification execution in the major class and the minor class. To combat class imbalance, HSMOTE creates synthetic data, and optimization algorithms zero in on the most important aspects to include. The model’s overall effectiveness is highly dependent on how these two processes interact with one another. For example, overfitting or underfitting might occur if there is an imbalance between the quantity of synthetic data created by HSMOTE and the number of features that were chosen. Inadequate synthetic data could fail to adequately balance the classes, affecting classification accuracy, while an excess of synthetic data without appropriate feature selection might add noise. Similarly, optimization techniques like FWDFA, AEHO, and FWGWO might hurt model performance if they are either strict or too lax in feature selection, leading to the loss of useful features or the retention of irrelevant ones. Consequently, getting the greatest outcomes requires finding the appropriate balance between HSMOTE and various optimization strategies.

**Fig. 3:**
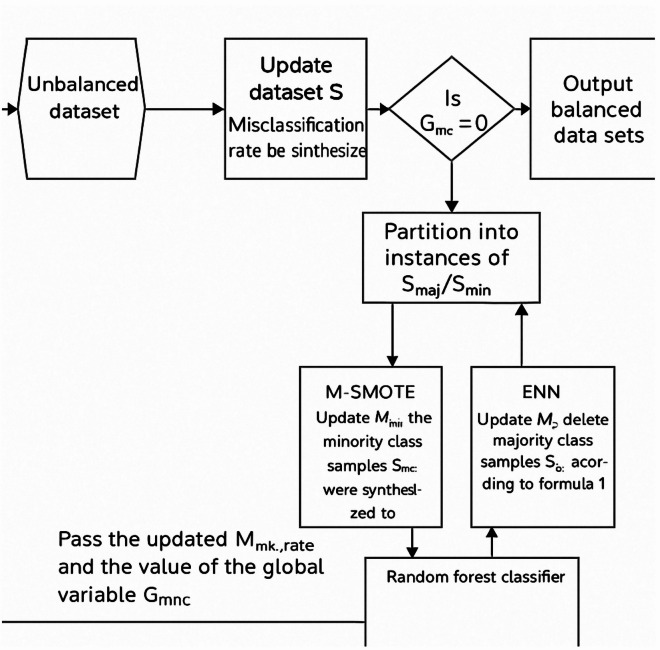
MSMOTE-kNN oversampling procedure.

This means that the iterative process should be terminated once the accuracy index decreases. The following is the MSMOTE-kNN flow chart as shown in Fig. 3:

The process of iteratively OS and US until sample is completely balanced is as shown in Fig. 3. By including some genuine samples in the min class and preserving the useful information of the maj class samples. The above algorithm, therefore, removes the noising and redundant data for maj class samples while gaining balancing sampling. MSMOTE-kNN is defined with the following steps as shown in the algorithm 3.

**ALGORITHM 3:**
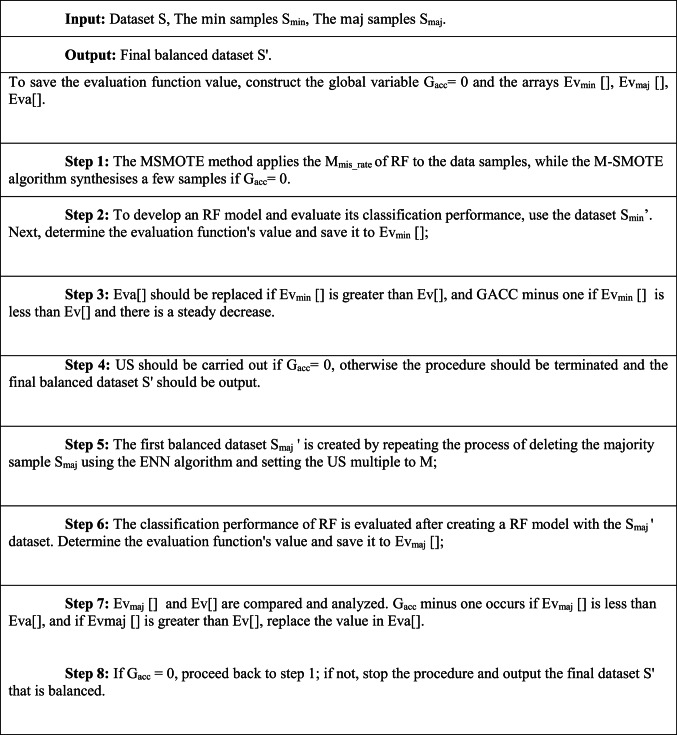
MSMOTE-kNN

### Optimization Ensemble Feature Selection Model (OEFSM)

An efficient ML technique is EL. Several selection techniques can be combined to achieve this. When compared to FS methods, the OEFSM exhibits superior stability and resilience. Combine the outcomes of the three approaches in this section.

**FW(Fuzzy Weight) GWO:** To choose the optimal attributes from the dataset, grey wolves (GW) are taken into consideration based on their amazing hunting skills and ability to capture their prey. Beta (β), Alpha (α), Delta (δ), and Omega (ω) are the four levels that this algorithm considers to reflect the GWO leadership hierarchy. In FS, α, which can be either female or male, stands for pack leaders when they make decisions about things like fishing, sleeping, and wake-up times. In FS, the candidates’ outstanding solutions are estimated to be ω^30^.

**Adaptive Elephant Herding Optimization (AEHO):** The primary functions of the model are initialisation, evaluation, transformation, and iteration. The process of initialising the arbitrarily produced population of n wolves or Search Agents (SA) is the first step. The length $d$ is equivalent to the number of features in the actual dataset, and each SA is associated with and indicates a desirable solution. Equation (5) sets the FF of the produced solutions assessment to balance the objectives.

5 $${\text{fitness}} = \upalpha \upgamma _{R} \left( {\text{D}} \right) +\upbeta \frac{{\left| {\text{S}} \right|}}{{\left| {\text{D}} \right|}}$$

Here, the EDDCM system’s error rate is represented by ${\upgamma }_{{\text{R}}} \left( {\text{D}} \right)$. The length of the chosen subset feature cardinality is denoted by |S|. The cardinality of every feature in the dataset is $\left| {\text{D}} \right|$. The evaluation function was used to determine the weight parameters ${\upalpha }$ and ${\upbeta }$, which represent the value of the accurate classification and the chosen subset length feature, ${\upalpha } \in { }\left[ {0,1} \right]$ and ${\upbeta } = 1 - {\upalpha }$. To facilitate learning between the pack of wolves and a particular wolf, the AEHO algorithm also incorporates the location data of the optimal, second-best wolves, as well as the location information of the wolf pack’s third-best solution, when updating the position. The clan updating operator with FW serves as the basis for the AEHO’s position updating procedure^31^.

**FW(DFA) Dragonfly Algorithm (FWDFA):** The dynamic swarming behaviors of dragonflies in nature were used as the model for the DFA. The two necessary stages of optimization, exploration and exploitation, are reflected in the dynamic swarming behaviors. Five factors alignment, separation, food factor, cohesion, and adversary factor, are used to guide the dragonflies during the exploration and exploitation stages for selecting the best features from the dataset. To select the best features from the dataset, the following criteria are controlled: alignment weight (afw), separation weight (sfw), food factor (ffw), cohesion weight (cfw), enemy factor (efw), and the inertia weight (wfw).

To achieve low alignment and high cohesiveness during the exploitation phase, and high alignment and low cohesion during the exploration phase, the factor weights are adjusted accordingly.

The following formula given in Eq. (6) is used to determine a dragonfly’s (DF) separation factor from other dragonflies in the neighborhood:

6 $${\text{S}}_{{\text{i}}} = - \mathop \sum \limits_{{{\text{j}} = 1}}^{{\text{N}}} {\text{X}}_{{\text{i}}} - {\text{X}}_{{\text{j}}}$$

As in Eq. (6), the current DF feature position X_i_. The jth neighbor’s feature position is denoted by X_j_. N is the number of DF that are nearby. The alignment factor, which is computed as follows, is used to match one DF velocity to that of other DF in the neighborhood.

7 $${\text{A}}_{{\text{i}}} = \frac{{\mathop \sum \nolimits_{{{\text{j}} = 1}}^{{\text{N}}} {\text{V}}_{{\text{j}}} }}{{\text{N}}}$$

As shown in Eq. (7), N is the number of DF that are nearby and V_j_ is the velocity of the jth neighbour. The following formula is used to determine the cohesion factor towards the neighborhood’s centre of mass:

8 $${\text{C}}_{{\text{i}}} = \frac{{\mathop \sum \nolimits_{{{\text{j}} = 1}}^{{\text{N}}} {\text{X}}_{{\text{j}}} }}{{\text{N}}} - {\text{X}}_{{\text{i}}}$$

As shown in Eq. (8), N is the number of DF that are nearby, and X_j_ is the feature position of the jth neighbour. The attraction of a DF to an optimal selection of features is known as the “food factor,” and it is computed as follows:

9 $${\text{F}}_{{\text{i}}} = {\text{X}}^{ + } - {\text{X}}_{{\text{i}}}$$

As inferred in Eq. (9), the food source’s feature position is represented by ${\text{X}}^{ + }$. The DF ability to divert attention from an enemy is known as the enemy factor, and it is determined by

10 $${\text{E}}_{{\text{i}}} = {\text{X}}^{ - } - {\text{X}}_{{\text{i}}}$$

As found in Eq. (10), the enemy’s feature position is shown by ${\text{X}}^{ - }$. The feature location of the ADF in a Search Space (SS) is updated and motions are simulated using two vectors: a feature location vector (X) and a step vectors (∆X). The definition of the ∆X is as follows:

11 $$\Delta {\text{X}}_{{\text{i}}}^{{{\text{t}} + 1}} = \left( {{\text{sf}}_{{\text{w}}} {\text{S}}_{{\text{i}}} + {\text{af}}_{{\text{w}}} {\text{A}}_{{\text{i}}} + {\text{cf}}_{{\text{w}}} {\text{C}}_{{\text{i}}} + {\text{ff}}_{{\text{w}}} {\text{F}}_{{\text{i}}} + {\text{ef}}_{{\text{w}}} {\text{E}}_{{\text{i}}} } \right) + {\text{wf}}_{{\text{w}}} \Delta {\text{X}}_{{\text{i}}}^{{\text{t}}}$$

As in Eq. (11), the separation FW in this case is ${\text{sf}}_{{\text{w}}}$. The ith DF separation is denoted by ${\text{S}}_{{\text{i}}}$. The alignment FW is denoted by ${\text{af}}_{{\text{w}}}$. The alignment of the ith DF is A_i_. The cohesion FW is denoted by ${\text{cf}}_{{\text{w}}}$. The ith dragonfly’s cohesion is denoted by ${\text{C}}_{{\text{i}}}$. The food factor’s FW is denoted by ${\text{ff}}_{{\text{w}}}$. The ith DF food factor is denoted by ${\text{F}}_{{\text{i}}}$. The FW of the enemy factor is denoted by ${\text{ef}}_{{\text{w}}}$. The ith dragonfly’s enemy factor is ${\text{E}}_{{\text{i}}}$. t is the iteration numbers, and ${\text{wf}}_{{\text{w}}}$ is the inertia FW. Following the step vector calculation, the dragonfly’ feature position is modified in the following way:

12 $${\text{X}}_{{\text{i}}}^{{{\text{t}} + 1}} = {\text{X}}_{{\text{i}}}^{{\text{t}}} + \Delta {\text{X}}_{{\text{i}}}^{{{\text{t}} + 1}}$$

As shown in Eq. (12), a radius is assumed around each ADF to take into account its neighbours. To obtain an ideal FS solution from a single dynamic swarm that will converge towards the global optimum solution, all of the DF will unite during the final optimisation stage. When they have no neighbours, they move through the feature selection SS using the Lévy flight (LF) mechanism. The following is the definition of the feature position update in this scenario:

13 $${\text{X}}_{{\text{i}}}^{{{\text{t}} + 1}} = {\text{X}}_{{\text{i}}}^{{\text{t}}} + {\text{Levy}}\left( {\text{d}} \right) \times {\text{X}}_{{\text{i}}}^{{\text{t}}}$$

As shown in Eq. (13), where d is the dimension of the feature location vector and t is the number of current iteration. Until the end criterion is satisfied, each DF step vector and feature position vectors are changed in each iteration. Algorithm 4 provides the DF algorithm pseudocode as shown in the algorithm 4.

**ALGORITHM 4:**
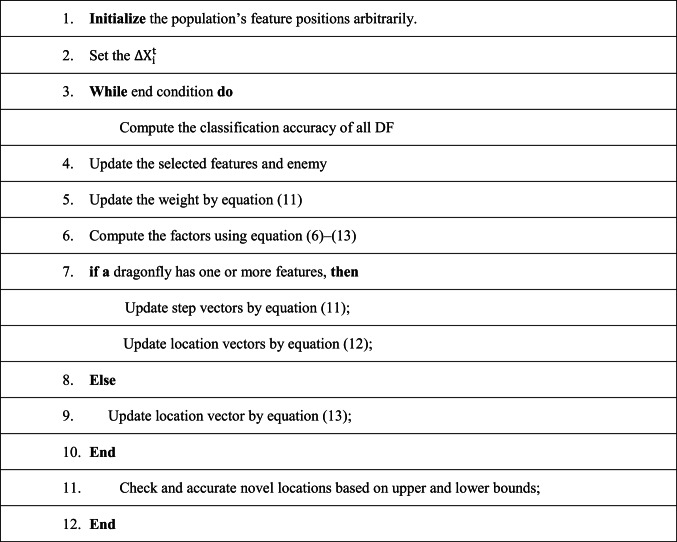
FUZZY WEIGHT DRAGONFLY ALGORITHM (FWDFA)

**Aggregation strategy:** The following is a detailed explanation of the methodology: (1) To acquire numerous sorted optimal feature subsets ${\mathbf{FS}}_{1} ,{ }{\mathbf{FS}}_{2} ... {\mathbf{FS}}_{{\mathbf{t}}}$, choose significant features using filter, wrapper, and embedded feature approaches, then sort features based on relevance.

(2) The significance of every feature j in the ${\mathbf{FS}}_{{\mathbf{i}}}$ is normalised with (n − j)/n (there are n features overall) to generate the feature weight sets of the ith FS technique ${\mathbf{Wg}}_{{\mathbf{i}}} = { }\left\{ {{\mathbf{wg}}_{1}^{{\text{i}}} ,{\mathbf{wg}}_{2}^{{\text{i}}} { }.{ }.{ }.{\mathbf{wg}}_{{\mathbf{n}}}^{{\text{i}}} } \right\}$. (3) Using a geometric mean aggregation approach, sort the n features according to the overall weight of every feature in the collection ${\mathbf{FS}}_{1} ,{ }{\mathbf{FS}}_{2} ... {\mathbf{FS}}_{{\mathbf{t}}}$. The sorted feature sequence ${\mathbf{Wg}}$ is then obtained. (4) The optimal feature subset is created by selecting the top th% of features from the sorted feature series based on the threshold th. (5) A classifier is used to confirm the HEFSM method’s performance based on the optimal feature subset.

### Ensemble Dynamic Classifier Model (EDDCM)

To maintain a dynamic pool of classifiers, including Density Weight CNN (DWCNN), Density Weight Long Short-Term Memory (DWLSTM), and Weight Autoencoder (WAE), the Ensemble Deep Dynamic Classifier Model (EDDCM) has been proposed, focusing on Accuracy and Diversity. The dynamic ensemble selection is used to choose classifiers based on diversity and accuracy. To determine whether the classifiers in the dynamic pool are representative of the current idea, they are prequentially tested on the current instance in the dataset. The dynamic size pool of classifiers is trained on the same instance.

A new classifier is trained using the most recent instance of data. To update the whole classifier pool, the classifier is then utilised. If the ensemble as a whole makes a mistake in its global forecast or concept drift, diverse classifiers that are representative of the present model are selected. A predefined parameter regulates the size of the dynamic pool of classifiers.

are strapped for cash but yet need

**Density Weight Convolutional Neural Network (DWCNN)**

To attain great performance, CNN employs a deep architecture with several layers and weights. With the suggested DWCNN approach, a substantially smaller number of sampled weights is used to calculate the weight values using the kernel density function. The dataset’s index with the value closest to the rising accuracy is transferred to the original weights. NN weights and the DWCNN classifier preserve the initial performance. output y, Feature Vectors (FV) z1 and z2, and input dataset (x). Using the output of the preceding layer z_i−1_as input, each layer i generates an output classification result ${\text{z}}_{{\text{i}}} \in {\mathbb{R}}^{{{\text{m}}_{{\text{i}}} \times {\text{n}}_{{\text{i}}} \times {\text{c}}_{{\text{i}}} }}$, also known as a feature. The layer input z_i−1_and the layer output z_i_ can have different dimensions.

The output classification results y are produced by the final layer, which uses the input dataset x as the first layer z_0_. There can be more than one operation in each layer. After convolving each input feature’s channel with a different filter, adding a constant value (weight (w) and bias (b)) to the classification results, and then adding the convolved dataset feature by feature, the common layer design applies a non-linear operation to each feature.

The capability of backpropagation (BP) enables the use of efficient gradient-based optimisation approaches. Because it accurately and efficiently computes partial gradients of the error concerning the filters and b for several commonly used error metrics^34^.

One parametric density estimation method that uses the kernel function to address the CNN classifier’s weight generation issue is Kernel Density Estimation (KDE). The kernel function is subject to two general conditions. The KDE creates a probability density function (PDF) about the initial weights using weights from a CNN that has already been trained. It is possible to sample data that reflects the original weights’ features by utilising this PDF. As per the Eq. (14), the smoothing parameter is h, which regulates the dataset size x. Based on their proximity, the kernel, or function K, determines the weight assigned to the observations x_i_ at each sample x. More effective quantisation processes are now possible because there are fewer sampled data points than there were original weights.

14 $${\text{f}}\left( {\text{x}} \right) = \frac{1}{{{\text{nh}}}}\mathop \sum \limits_{{{\text{i}} = 1}}^{{\text{n}}} {\text{K}}\left( {\frac{{{\text{x}}_{{\text{i}}} - {\text{x}}}}{{\text{h}}}} \right)$$

By aggregating all of these kernel functions, including RBF and Gaussian, and dividing the result by the overall number of data points, the kernel function f(·) for the observed data x can create the PDF. The features of the observed data are reflected in the calculated PDF. From a distribution of seen data, fresh data can be sampled using this function.

**Density Weight Long Short-Term Memory (DWLSTM):** DWLSTM is a novel structure that is proficient in learning long-term dependencies between the input and output. It looks for the prior output of the hidden state ht − 1 at every time step and the current input xt while processing data^35^. Cells are repeating modules that are provided for every time step in the design of LSTM. A set of gates that depend on the prior ht − 1 and an input of the current time-step xt control the output of a module at each time step, as shown by the following output gate ot, input gate it, and forget gate ft.

The current hidden state h_t,_ and the current memory unit c_t_ are updated based on the combined action of these gates. All of the vectors in this structure share a similar dimension, which is represented by the d in LSTM. LSTM on the convolution layer (CL) was selected to learn this dependency in the higher-level feature sequence since it is specifically made to learn time-series (TS) data for long-term dependencies.

**Fig. 4:**
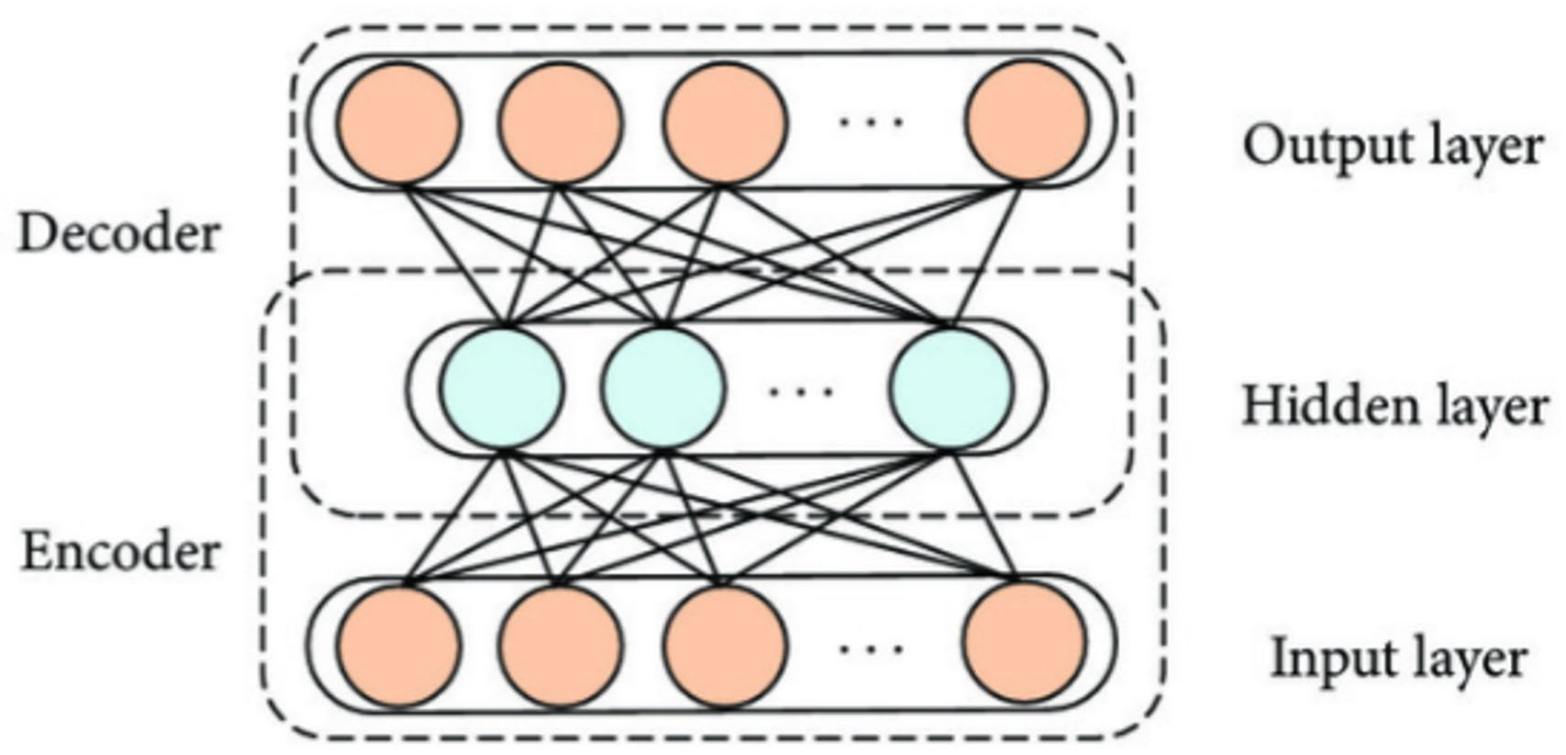
Architecture of the Weighted Autoencoder (WAE).

**Weight Autoencoder (WAE):** As seen in Fig. 4, a type of unsupervised learning structure named AE that consists of a Hidden Layer (HL), an Input Layer (IL), and an Output Layer (OL)- the 3 layers.

As shown in Fig. 4. The two parts of the training procedure for the AE are identical; one is known as the encoder (E), which maps the input data to a hidden depiction, and the second is called the decoder (D), which rebuilds the input data from the corresponding hidden depiction. The OL’s D vector is $\hat{x}_{n}$, and h_n is the hidden E vector derived from $x_{n}$, given the unlabelled input dataset $\left\{ {x_{n} } \right\}_{n = 1}^{N}$, Here, $x_{n} \in R^{m \times 1}$. Thus, the subsequent is the E process:

15 $${\text{h}}_{{\text{n}}} = {\text{f}}\left( {{\text{W}}_{1} {\text{x}}_{{\text{n}}} + {\text{b}}_{1} } \right)$$

As inferred from Eq. (15), where ${\text{W}}_{1}$ is the encoder’s weight matrices, ${\text{b}}_{1}$ is the bias vectors, and $f$ is the encoding functions.

The following is the description of the D procedure:

16 $$\hat{x}_{n} = {\text{g}}\left( {{\text{W}}_{2} {\text{h}}_{{\text{n}}} + {\text{b}}_{2} } \right)$$

As shown in Eq. (16), ${\text{b}}_{2}$ is the bias vectors, ${\text{W}}_{2}$ is the decoder’s weight matrices, and g is the decoding functions. The AE parameter sets are tuned to reduce the reconstruction error as in Eq. (17).

17 $$\phi \left( \Theta \right) = \arg \mathop {\min }\limits_{{{\uptheta ,\theta^{\prime}}}} \frac{1}{{\text{n}}}\mathop \sum \limits_{{{\text{i}} = 1}}^{{\text{n}}} {\text{L}}\left( {{\text{x}}^{{\text{i}}} , {\hat{\text{x}}}^{{\text{i}}} } \right)$$

1. Get the acquired feature vector (FV) by training the primary AE with input information;
2. Until the training process is finished, the FV from the prior layer is utilized as the input for the next layer.
3. To achieve FT, the BP method is utilised to update the weights using a labelled training set and minimise the cost function after all of the hidden layers (HL) have been trained.

**Dropout:** It has been demonstrated that dropout is an efficient technique for lowering overfitting in NN training. A small training set is always the cause of the overfitting issue. On the test set, this approach will lead to poor accuracy. During training, dropout can cause the HL neurons to lose power randomly.

**ReLU**: When training error spreads to forward layers for conventional activation functions (AF) (sigmoid and hyperbolic tangent functions), the gradients rapidly diminish. In Eq. (18), the ReLU function is presented as in Eq. (18).

18 $$f_{r} \left( x \right) = \max \left( {0,{\text{x}}} \right)$$

**Ensemble approach:** To manage concept drift, shorten the time it takes for new concepts to converge, and effectively manage various drift types, EDDCM offers both active and passive methods. When a classifier’s accuracy and diversity drop below a predetermined threshold (T), EDDCM eliminates the classifiers with the lowest Accuracy and diversity from the dynamic pool (DP) to apply a passive strategy. Both passive and active methods preserve the heterogeneity of the ensemble while reducing overheads and computational costs by limiting the ensemble size from expanding infinitely. Drift detection is used to implement the active approach, and EDDCM resets the entire learning system when a drift detection mechanism indicates that the ensemble’s global prediction is inaccurate. To identify concept drift and warnings, the drift detection systems use the predictions produced by the base classifiers. The pupil with the lowest accuracy and equivalent variety is detached from the DP as shown in the algorithm 5.

**ALGORITHM 5:**
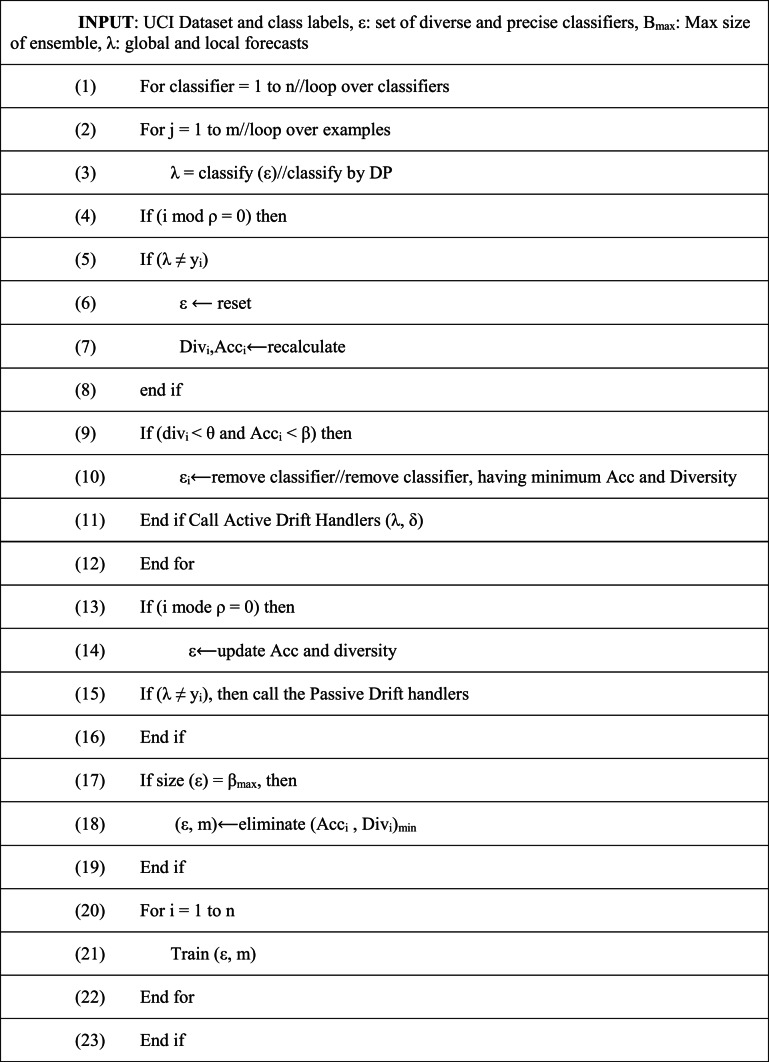
EDDCM

Companies dealing with massive datasets and issues like class imbalance, feature selection, and dynamic classification jobs might greatly benefit from the suggested methodology. Having a good sense of when and how to anticipate unusual occurrences (such as fraud detection or illness diagnosis) is vital in industries such as healthcare, banking, and online commerce. Because it integrates HSMOTE, OEFSM, and EDDCM, the model successfully tackles the problem of class imbalance. This is especially prevalent in sectors like fraud cases and unusual medical diseases, which disproportionately affect minority classes. Due to its scalability and computing efficiency, the model is perfect for small enterprises that do not have the resources of bigger organizations. Metaheuristic algorithms enable small-scale organizations to achieve competitive prediction performance without requiring extensive computational resources. They do this by using dynamic ensemble classification and feature selection. To put it another way, the concept is a godsend for startups, mom-and-pop clinics, and other tiny businesses who are strapped for cash but yet need fast decision-making methods as shown in the algorithm 6.

**ALGORITHM 6:**
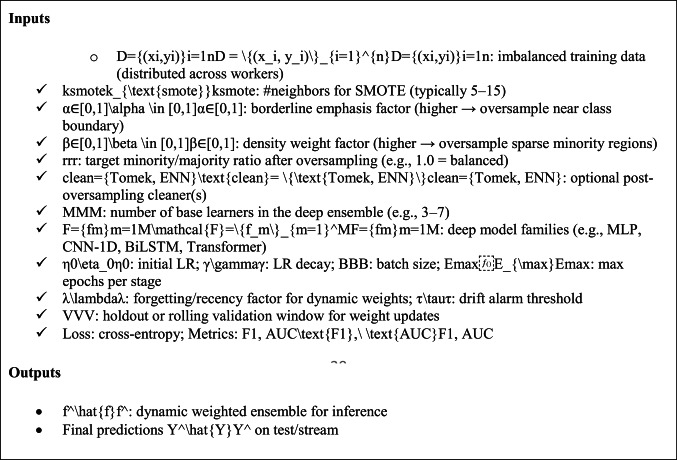
HSMOTE–EDDCM (End-to-End Big-Data Pipeline)

In algorithim 6, The proposed HSMOTE–EDDCM pipeline is designed to address the twin challenges of class imbalance and high-dimensional big data classification. The begin with the imbalanced dataset D = {(xi,yi)}i = 1nD = \{(x_i, y_i)\}_{i = 1}^nD = {(xi,yi)}i = 1n, which may be distributed across multiple computing nodes for scalability. The parameter ksmotek_{\text{smote}}ksmote defines the number of nearest neighbors used in the synthetic oversampling process. Two weighting factors—α\alphaα for borderline emphasis and β\betaβ for density sensitivity—control how strongly the algorithm oversamples instances near class boundaries or in sparse minority regions, respectively. The target balance ratio rrr specifies the desired minority-to-majority proportion after oversampling. An optional cleaning stage, controlled by the clean parameter, can apply methods such as Tomek link removal or Edited Nearest Neighbor (ENN)filtering to eliminate noise and overlapping samples. The ensemble stage uses MMM base learners from a predefined set of deep learning architectures F = {fm}m = 1MF = \{f_m\}_{m = 1}^MF = {fm}m = 1 M, which may include MLPs, CNNs, BiLSTMs, or Transformers. Training hyperparameters include the initial learning rate η0\eta_0η0, its decay factor γ\gammaγ, the batch size BBB, and the maximum epochs per stage Emax⁡E_{\max}Emax. The dynamic weighting of ensemble members is controlled by a recency factor λ\lambdaλ, which gives more weight to recent performance, and a drift threshold τ\tauτ that triggers adaptation if data distribution shifts are detected. The validation set VVV is used both for weight updates and for early stopping decisions. Model optimization uses cross-entropy loss, while evaluation focuses on F1-score and AUC to handle imbalanced scenarios. The **outputs** are a trained, dynamically weighted ensemble model f^\hat{f}f^ that adapts to changing data conditions, and the final predicted labels Y^\hat{Y}Y^ for test or streaming input data, ensuring robust, balanced classification in large-scale, real-world applications.

## Results and discussion

All tests were conducted using the 64-bit version of Windows 10 Pro; the processor was an Intel(R) i7-8550U with 16 GB of RAM and a 1.80–1.99 GHz core. MATLAB (2014a) is used to apply all algorithms. The suggested and current FS techniques are researched and evaluated using well-known datasets in the field of FS problems to verify their efficacy, efficiency, and strength. Four benchmark datasets (see Table 2) from the UCI ML Repository (Breast Cancer Data) are used to analyse FS techniques .

**Table 2:** Dataset description.

No.	Dataset	Instances	No. of features	Classes
1	KC1	2110	21	2
2	WDBC	569	31	2
3	SCENE	2407	299	2
4	SEGMENT	2310	20	7

Differentiating between benign and malignant tumors is greatly facilitated by the chosen features, which include mean compactness, mean smoothness, mean texture, and mean radius. To classify tumors accurately, these criteria capture crucial tumor properties. For example, because malignant tumors are often bigger, the mean radius is an important element in predicting malignancy since it gives insights into the overall size of the tumor. The uneven architecture of malignant tumors causes their mean texture, which quantifies the variation in pixel intensities, to be larger. In a similar vein, the smoothness of the tumor’s border is reflected in its mean smoothness; malignant tumors often have more uneven and rough margins than those that are benign. The feature selection procedure increased computational efficiency and classification accuracy by concentrating on these important characteristics and ignoring less useful ones, such as area or perimeter. This allowed the model to better distinguish between benign and malignant instances.

The CNN-BiLSTM-GRU ensemble with dynamic voting architecture is computationally intensive and can pose challenges in terms of scalability, particularly in environments requiring real-time or streaming data processing. For instance, a typical training session for this model could take several hours, depending on the dataset size (e.g., 500,000 data points), and inference latency might be in the range of 100-300 ms per sample on a standard GPU setup (e.g., NVIDIA RTX 2080 Ti).

Features in the BC Wisconsin data collection are identified using digital images of fine needle aspirates (FNAs) taken of breast masses. They delineate the distinctive characteristics of the picture’s cell nuclei. Donor, source, clinical tests, identification number, grade distribution, and diagnosis (malignant = M, benign = B) are all part of it. See Table 2 for a list of the ten real-valued functions that were found for each cell nucleus: radius, texture, area, perimeter, compactness, concavity, concave points, symmetry, and fractal dimension. The “worst” or greatest value, regular error, and mean are all measured for each property. In this classification stage, the provided data series comprise 80% training data and 20% evaluation data.

The CKD data source is composed of 400 cases with 25 features. This dataset can be obtained from the hospital within about eight weeks and is also used to evaluate CKD. The scene dataset is an Image classification challenge where each image is assigned a label such as Beach, Mountain, Field, or Urban. From a database of seven outdoor images, segment instances were chosen at random. For every pixel of the images, manual segmentation is performed. The use of each instance has a 3 × 3 region. Table 3 shows the parameter settings.

**Table 3:** Parameter setting.

Method/model	Parameter setting
HSMOTE	Neighbors (k): 5, Sampling Ratio: 1.0, Distance Metric: Euclidean
OEFSM	FWDFA: Population Size: 50, Max Iterations: 100, Convergence Tolerance: 0.001
	AEHO: Population Size: 40, Max Iterations: 150, Herding Strength: 0.8
	FWGWO: Population Size: 60, Max Generations: 100, Alpha Coefficient: 1.5
EDDCM	DWCNN: Number of Layers: 3, Filter Size: 3 × 3, Dropout Rate: 0.5
	DWBi-LSTM: Hidden Units: 128, Dropout Rate: 0.3, Epochs: 50

The number of true positives for a category is divided by the total number of items identified as belonging to the positive class to determine its precision. Equation (19) provides its definition.

19 $${\text{Precision}} = \frac{{{\text{True Positive}}\left( {{\text{TP}}} \right){ }}}{{{\text{True Positive}}\left( {{\text{TP}}} \right) + {\text{False Positive}}\left( {{\text{FP}}} \right)}}$$

The number of TP divided by the total number of samples that belong to the positive class is how recall is defined in this instance. Equation (20) is expressed below,

20 $${\text{Recall}} = \frac{{{\text{True Positive}}\left( {{\text{TP}}} \right){ }}}{{{\text{True Positive}}\left( {{\text{TP}}} \right) + {\text{False Negative}}\left( {{\text{FN}}} \right)}}$$

The combination of precision and recall is known as the F-measure. Equation (21) is formulated as,

21 $${\text{F}} - {\text{Measure}} = \frac{{2 \times {\text{precision}} \times {\text{recall}}}}{{{\text{precision}} + {\text{recall}}}}$$

The ratio of accurate predictions to all predictions is known as classification accuracy. Equation (22) is frequently used to describe it.

22 $${\text{Accuracy }} = \frac{{{\text{TP}} + {\text{TN}}}}{{{\text{TP}} + {\text{TN}} + {\text{FP}} + {\text{FN}}}}$$

Four FS techniques CWOA, GWO, PSO-GWO, and the suggested HEFSM framework) were used in combining experiments with classifiers and four datasets. The results presents Precision (P), Recall (R), f-measure, accuracy (Acc), and computation time.

The precision values in Table 4 highlight how well each classification method can correctly identify the positive instances across different datasets. For example, the PSO-GWO method shows a significant improvement in precision over CWOA and GWO, particularly on WDBC (98.15%) and Segment (97.35%), demonstrating the superiority of hybrid optimization methods in reducing false positives. The EDDCM consistently outperforms other models across all datasets, achieving precision values above 99% in all cases, indicating its robust performance in minimizing errors in predicting the minority class, especially with challenging datasets like SCENE and WDBC. This study reporting precision as “mean ± SD” (e.g., 95.6% ± 1.2%) will indicate not only the average performance but also the variability across different runs.

**Table 4:** Precision comparison of classification methods vs. datasets.

Dataset	Methods	KNN	SVM	DWCNN	DWLSTM	HEDCM	EDDCM
WDBC	CWOA	90.00	91.44	92.08	92.70	94.04	95.56
	GWO	93.99	94.41	95.71	95.98	97.01	98.45
	PSO-GWO	96.99	97.64	98.15	98.34	99.25	99.58
	HEFSM	97.99	98.37	98.51	99.01	99.44	99.89
KC1	CWOA	78.00	81.28	83.34	86.59	91.05	94.56
	GWO	79.47	82.91	84.56	87.40	91.75	93.25
	PSO-GWO	81.43	83.28	84.94	89.00	92.71	95.10
	HEFSM	83.62	85.6	86.55	90.77	93.98	95.78
SCENE	CWOA	88.12	90.37	91.72	93.19	94.80	96.00
	GWO	90.81	93.12	93.35	93.87	95.64	96.90
	PSO-GWO	84.56	93.88	94.48	96.11	96.44	97.17
	HEFSM	86.16	95.42	96.03	96.51	97.70	97.78
SEGMENT	CWOA	91.00	92.23	92.96	94.60	96.04	96.64
	GWO	94.33	95.50	96.07	96.72	97.69	98.15
	PSO-GWO	96.25	96.96	97.35	98.41	99.01	99.45
	HEFSM	97.24	98.05	98.54	98.99	99.37	99.73

In Table 5, recall measures the ability of the models to identify all relevant instances (true positives) correctly. PSO-GWO again outperforms CWOA and GWO, showing superior recall values, such as 97.55% for WDBC and 98.46% for Segment. Notably, EDDCM consistently exhibits the highest recall, reaching 99% in most cases, particularly on the WDBC and SCENE datasets. This suggests that EDDCM excels at identifying minority class instances, which is critical for imbalanced datasets where detecting rare events (e.g., fraud detection) is essential.

**Table 5:** Recall comparison of classification methods vs. datasets.

Dataset	Methods	KNN	SVM	DWCNN	DWLSTM	HEDCM	EDDCM
WDBC	CWOA	80.11	82.94	86.31	88.03	89.78	90.45
	GWO	82.01	84.34	90.22	91.81	96.81	97.56
	PSO-GWO	85.00	86.93	90.90	93.10	97.55	97.90
	HEFSM	86.02	88.19	92.49	94.71	98.52	99.01
KC1	CWOA	78.00	79.40	81.53	82.26	84.88	86.78
	GWO	80.14	81.50	82.46	83.36	85.93	87.00
	PSO-GWO	82.20	83.40	84.98	86.67	88.74	89.95
	HEFSM	84.51	86.02	87.88	89.63	91.89	93.17
SCENE	CWOA	89.00	89.94	91.46	92.55	94.67	95.61
	GWO	90.87	91.64	92.82	93.91	95.31	97.11
	PSO-GWO	91.66	92.50	93.83	95.02	97.10	98.34
	HEFSM	92.75	93.44	94.94	96.52	97.71	98.23
SEGMENT	CWOA	90.00	91.08	91.80	92.62	94.90	95.71
	GWO	93.75	94.44	95.57	96.00	96.44	97.16
	PSO-GWO	95.14	96.10	96.96	97.48	98.46	98.99
	HEFSM	96.23	96.94	97.32	98.15	99.05	99.61

Table 6 compares F-measure, which balances precision and recall. EDDCM consistently provides the highest F-measure, particularly in datasets like WDBC (99.56%) and Segment (99.70%), demonstrating its overall balanced performance. The PSO-GWO method also performs well, but the EDDCM method maintains a higher balance, showing its advantage in handling the trade-off between precision and recall. The results suggest that EDDCM not only reduces false positives but also minimizes false negatives, making it a highly reliable model across different datasets.

**Table 6:** F-measure comparison of classification methods vs. datasets.

Dataset	Methods	KNN	SVM	DWCNN	DWLSTM	HEDCM	EDDCM
WDBC	CWOA	85.05	87.30	89.30	90.47	92.02	94.10
	GWO	87.98	89.48	93.07	94.00	97.02	98.04
	PSO-GWO	90.99	92.39	94.63	95.83	98.51	99.15
	HEFSM	91.58	93.39	95.61	96.97	99.09	99.56
KC1	CWOA	78.00	80.45	82.54	84.53	88.07	90.18
	GWO	79.80	82.31	83.62	85.49	88.95	89.78
	PSO-GWO	81.81	83.45	85.07	87.99	90.83	91.32
	HEFSM	84.06	85.92	87.32	90.31	93.04	95.13
SCENE	CWOA	88.56	90.26	91.70	92.98	94.84	96.13
	GWO	90.84	92.49	93.19	94.00	95.58	97.17
	PSO-GWO	92.11	93.30	94.26	95.67	96.88	97.89
	HEFSM	93.45	94.54	95.59	96.62	97.81	98.26
SEGMENT	CWOA	90.50	91.76	92.49	93.72	95.58	96.67
	GWO	94.04	95.08	95.93	96.47	97.17	98.24
	PSO-GWO	95.69	96.64	97.26	98.05	98.84	99.16
	HEFSM	96.73	97.60	98.04	98.68	99.32	99.70

In terms of accuracy, EDDCM achieves the highest performance across all datasets, with values close to 99%. For instance, EDDCM achieves 99.89% accuracy on WDBC and 99.88% on Segment, indicating that it is effective at correctly classifying both majority and minority instances in imbalanced datasets, as in Table 7. The PSO-GWO method also delivers high accuracy but falls short compared to EDDCM. This suggests that the ensemble approach with dynamic voting in EDDCM enhances classification accuracy by leveraging the strengths of various classifiers.

**Table 7:** Accuracy comparison of classification methods vs. datasets.

Dataset	Methods	KNN	SVM	DWCNN	DWLSTM	HEDCM	EDDCM
WDBC	CWOA	91.00	92.14	92.69	93.42	94.72	95.89
	GWO	94.00	95.35	95.43	96.13	96.60	97.26
	PSO-GWO	97.00	97.87	98.36	98.64	99.30	99.78
	HEFSM	98.00	98.72	99.04	99.18	99.64	99.89
KC1	CWOA	78.00	79.19	80.84	82.34	84.88	86.67
	GWO	80.84	82.52	85.12	86.24	87.54	88.53
	PSO-GWO	82.84	85.05	85.89	86.57	88.52	89.05
	HEFSM	84.83	86.84	87.38	88.14	90.24	92.64
SCENE	CWOA	88.56	90.82	91.85	92.45	94.57	95.91
	GWO	90.82	92.23	92.45	93.15	95.72	96.46
	PSO-GWO	91.82	93.97	94.53	95.65	97.35	98.27
	HEFSM	92.85	94.10	95.01	95.51	98.74	99.26
SEGMENT	CWOA	91.00	92.5	92.86	93.97	95.46	96.80
	GWO	94.28	95.42	96.06	96.90	98.03	98.72
	PSO-GWO	96.24	97.43	97.91	98.35	99.14	99.80
	HEFSM	97.23	97.84	98.51	98.99	99.54	99.88

While EDDCM provides the best classification performance, it is essential to note its computation time in Table 8. It consistently outperforms other methods in terms of classification quality, but with an increased computational cost compared to KNN or SVM, which are faster but less accurate. For example, on the SCENE dataset, EDDCM requires significantly more computational time (46.23 s) than KNN (9.2 s). However, this trade-off between accuracy and computational time is common in ensemble DL models, and the results emphasize that EDDCM is more suitable for applications where high accuracy is more critical than computational efficiency, such as in fraud detection or medical diagnostics.

**Table 8:** Computation time comparison of classification methods vs. datasets.

Dataset	Methods	KNN	SVM	DWCNN	DWLSTM	HEDCM	EDDCM
WDBC	CWOA	9.2	8.18	7.11	6.54	5.68	5.10
	GWO	8.4	7.72	6.33	5.38	4.94	4.04
	PSO-GWO	7.5	7.05	5.72	5.02	4.14	3.67
	HEFSM	6.9	6.09	5.11	4.74	3.42	3.01
KC1	CWOA	12.15	11.17	9.94	7.95	6.96	5.45
	GWO	11.86	10.91	9.11	7.19	6.28	5.22
	PSO-GWO	8.87	7.80	6.55	5.74	4.87	4.11
	HEFSM	7.63	7.01	5.15	5.23	4.14	3.44
SCENE	CWOA	92	88.32	75.95	61.52	52.29	46.23
	GWO	86	76.54	68.12	53.81	41.43	38.90
	PSO-GWO	65	57.85	50.22	45.23	37.08	34.66
	HEFSM	52	47.54	45.57	39.64	33.69	29.67
SEGMENT	CWOA	15.82	14.55	13.09	12.04	10.59	9.12
	GWO	13.93	12.18	11.20	10.19	8.97	8.02
	PSO-GWO	10.75	9.87	9.09	7.98	6.62	5.90
	HEFSM	9.82	8.53	7.05	5.92	4.67	4.10

**Fig. 5:**
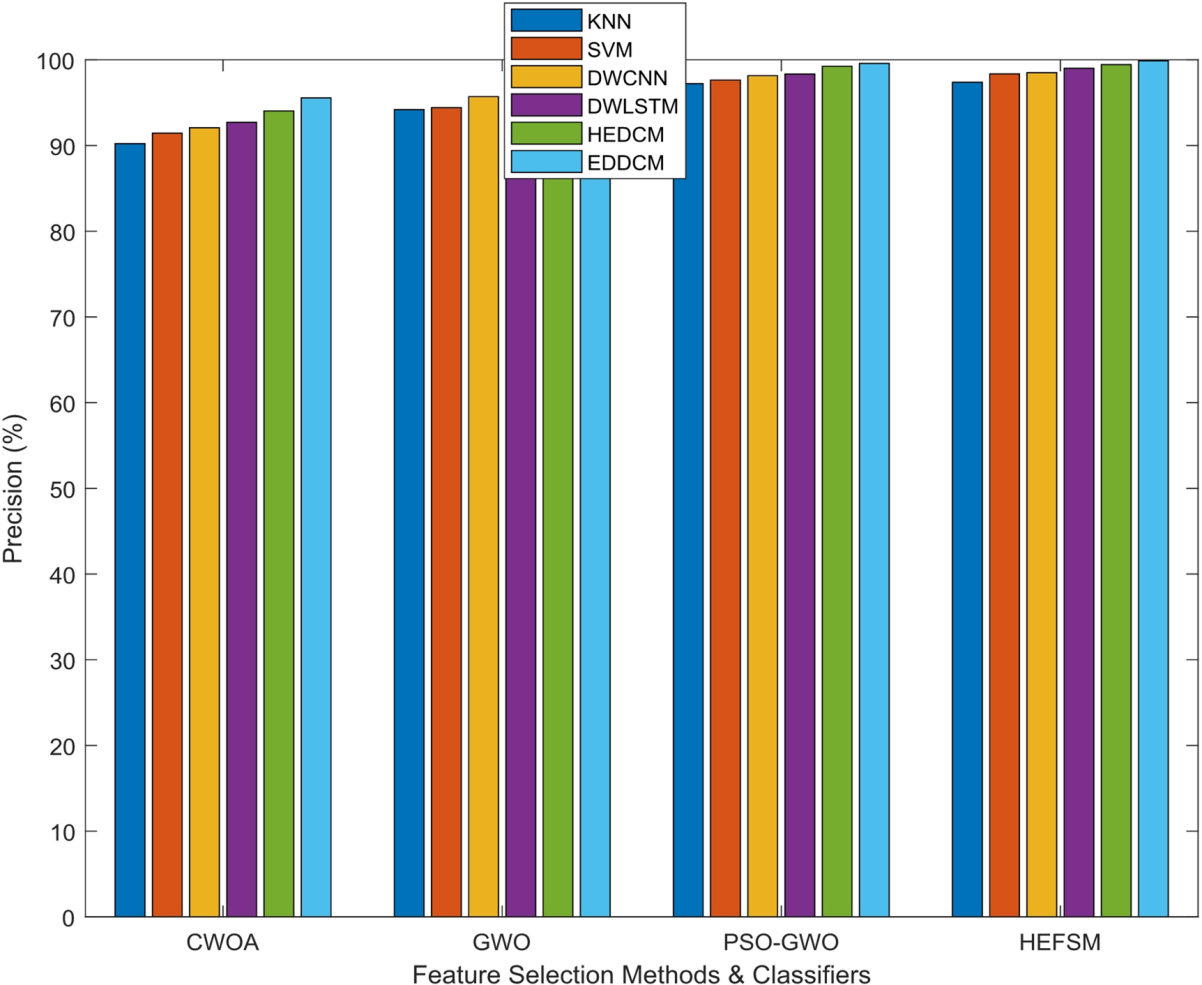
Precision comparison of feature selection methods and classifiers.

Precision results comparison of classifiers such as KNN, SVM, DWCNN, DWLSTM, HEDCM, and EDDCM is illustrated in Fig. 5. These classifiers are compared with four feature selection methods. The proposed algorithm yields a greater P outcome of 99.89%, whereas other approaches, including KNN, SVM, DWCNN, DWLSTM, and HEDCM, achieve lower precision results of 97.99%, 98.37%, 98.51%, 99.01%, and 99.44% on the WDBC dataset, as reported by HEFSM. Proposed EDDCM algorithm gives higher precision results of 95.56%, 98.45%, 99.58%, and 99.89% for CWOA, GWO, PSO-GWO, and HEFSM in the WDBC dataset.

**Fig. 6:**
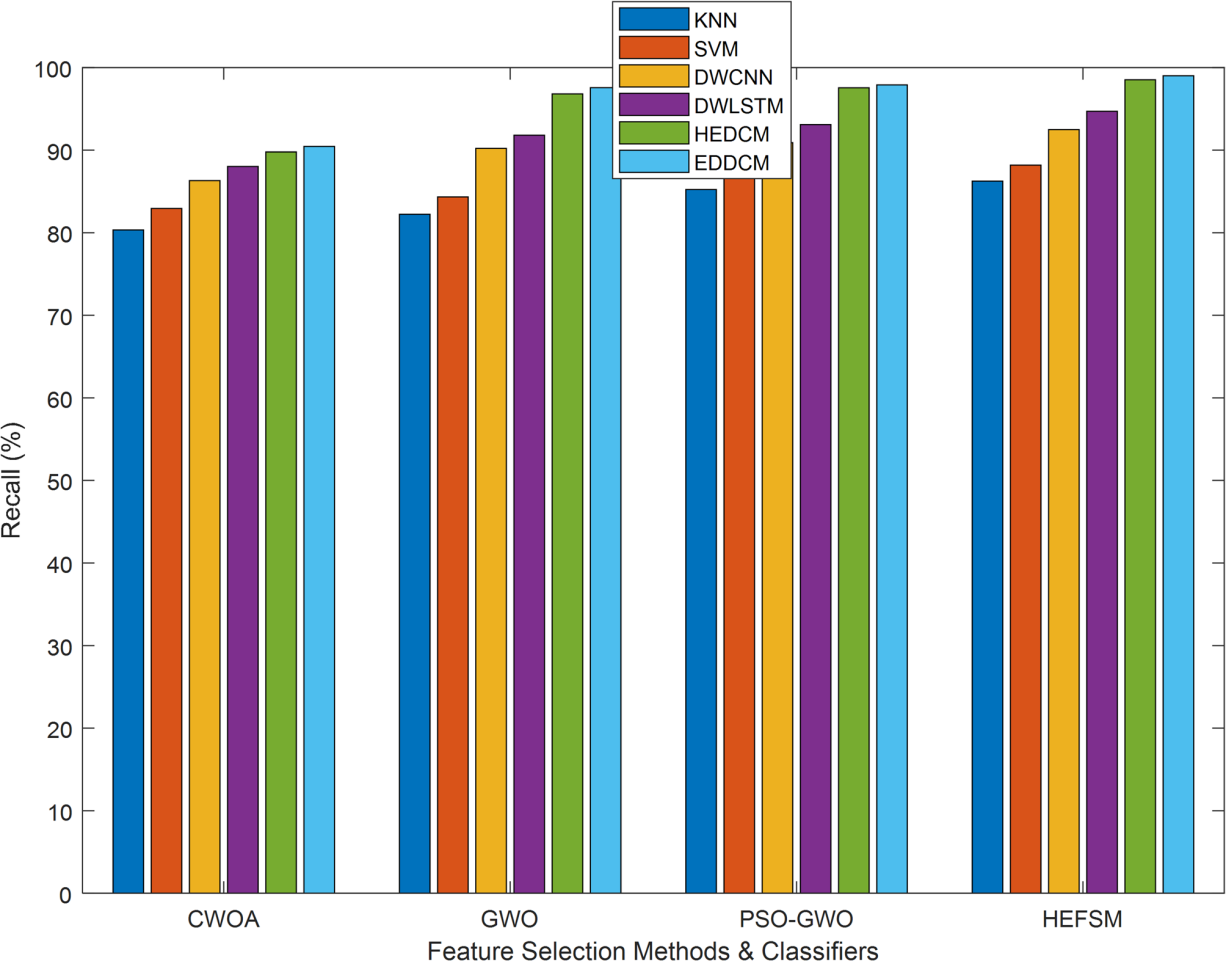
Recall comparison of feature selection methods and classifiers.

KNN, SVM, DWCNN, DWLSTM, HEDCM, and EDDCM are compared in terms of R outcomes, as shown in Fig. 6. The proposed algorithm yields a higher result of 99.01%, whereas other methods, such as KNN, SVM, DWCNN, DWLSTM, and HEDCM, produce lower results of 86.02%, 88.19%, 92.49%, 94.71%, and 98.52% on the WDBC dataset, as evaluated by HEFSM. Proposed EDDCM algorithm gives greater outcomes of 90.45%, 97.56%, 97.90%, and 99.01% for CWOA, GWO, PSO-GWO, and HEFSM in the WDBC dataset.

**Fig. 7:**
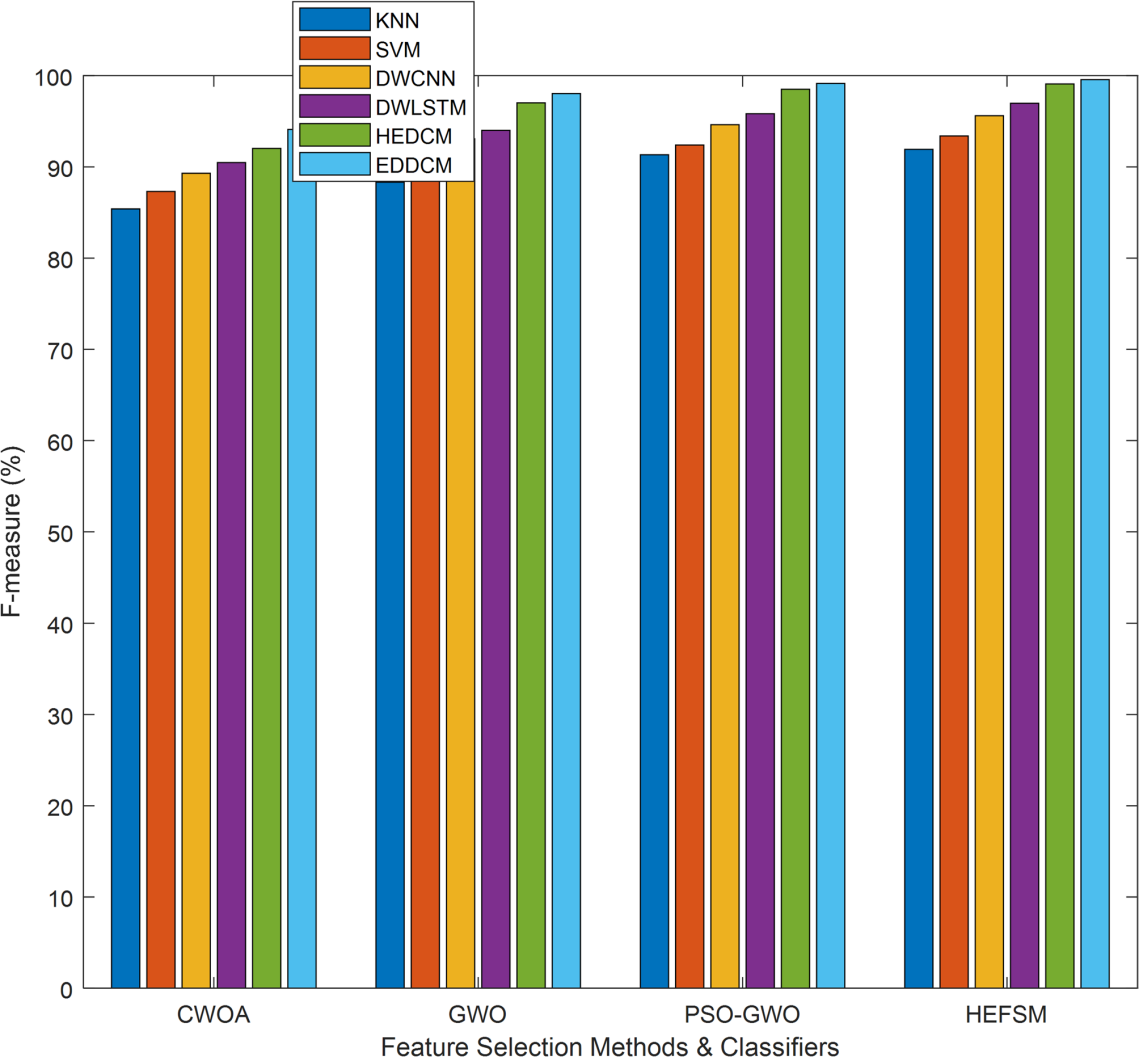
F-Measure analysis for feature selection and classification approaches.

F-measure results comparison of classifiers such as KNN, SVM, DWCNN, DWLSTM, HEDCM, and EDDCM is illustrated in Fig. 7. The proposed algorithm yields a higher result of 99.56%, whereas other approaches, such as KNN, SVM, DWCNN, DWLSTM, and HEDCM, achieve lower results of 91.58%, 93.39%, 95.61%, 96.97%, and 99.09% on the WDBC dataset of the HEFSM algorithm. Proposed EDDCM algorithm gives higher results of 94.10%, 98.04%, 99.15%, and 99.56% for CWOA, GWO, PSO-GWO, and HEFSM in the WDBC dataset.

**Fig. 8:**
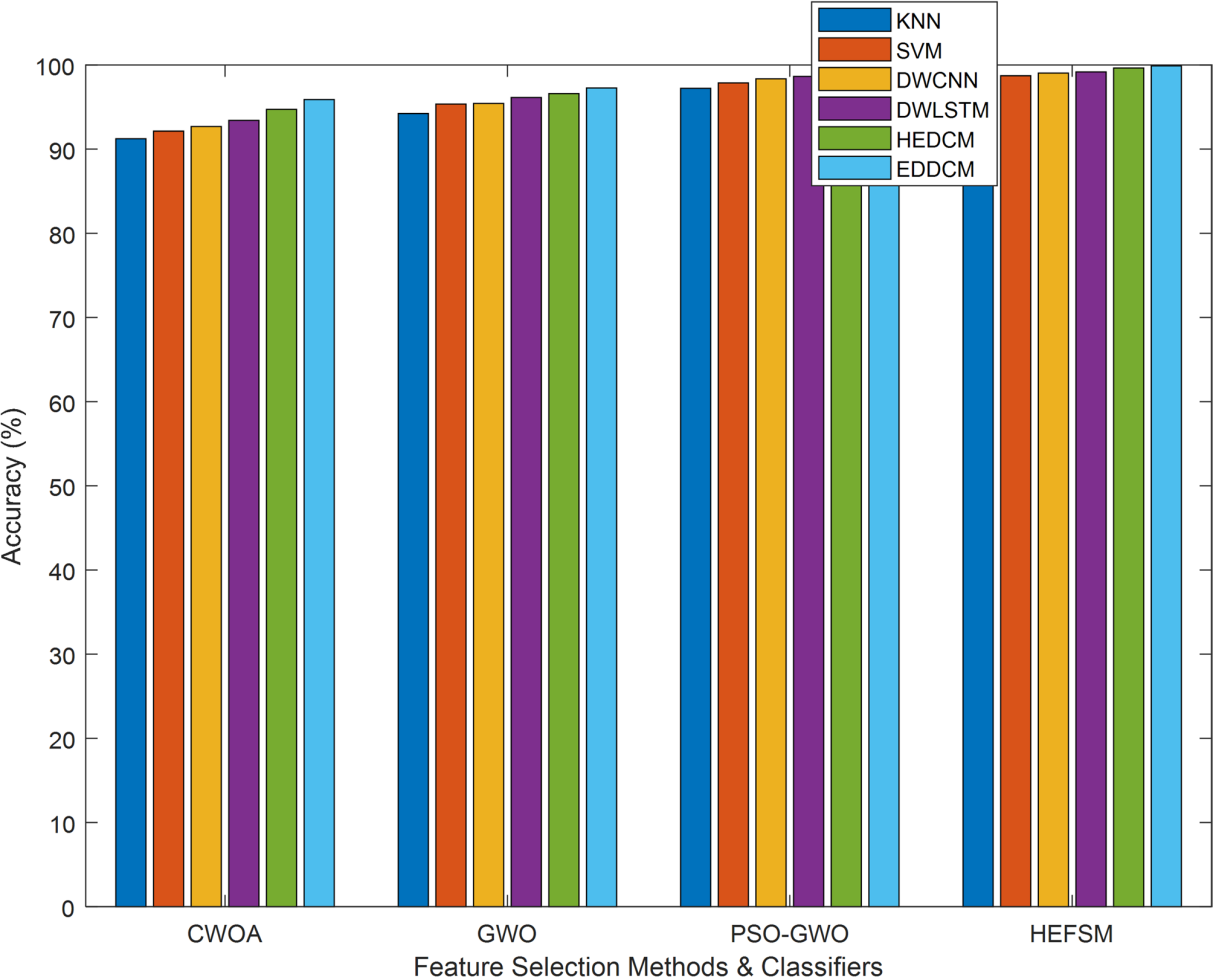
Accuracy analysis for different FS-classifiers combinations.

Four FS methods concerning classifiers via f-measure results are illustrated in Fig. 8. The proposed classifier yields an increased outcome of 99.89%, whereas other approaches, such as KNN, SVM, DWCNN, DWLSTM, HEDCM, and EDDCM, produce reduced results of 98.00%, 98.72%, 99.04%, 99.18%, and 99.64% on the WDBC dataset of the HEFSM algorithm. Proposed classifier shows highest results of 95.89%, 97.26%, 99.78%, and 99.89% for CWOA, GWO, PSO-GWO, and HEFSM algorithms in the WDBC dataset .

**Fig. 9:**
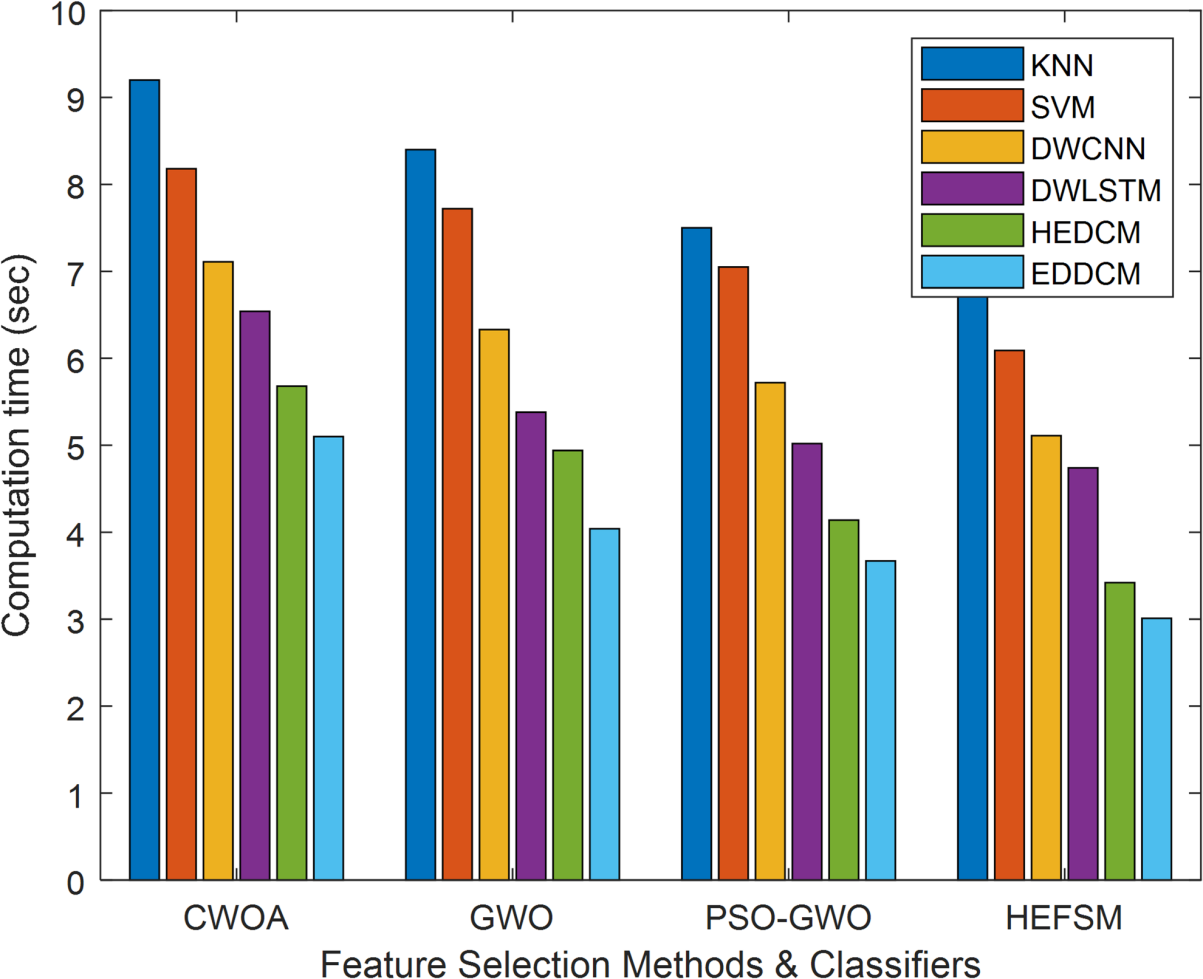
Computational time comparison for FS techniques and classifiers.

The comparison time between the suggested model and other algorithms concerning the FS methods is shown in Fig. 9. The suggested model took less than 3.01 s to compute, while other classifiers such as KNN, SVM, DWCNN, DWLSTM, HEDCM, and EDDCM took less time: 6.9, 5.11, 4.74, and 3.42 s, respectively, for the proposed FS technique. For the WDBC dataset, the HEFSM requires computation times of 5.10 s, 4.04 s, and 3.67 s, respectively, compared to the current FS methods, CWOA, GWO, and PSO-GWO algorithms.

**Fig. 10:**
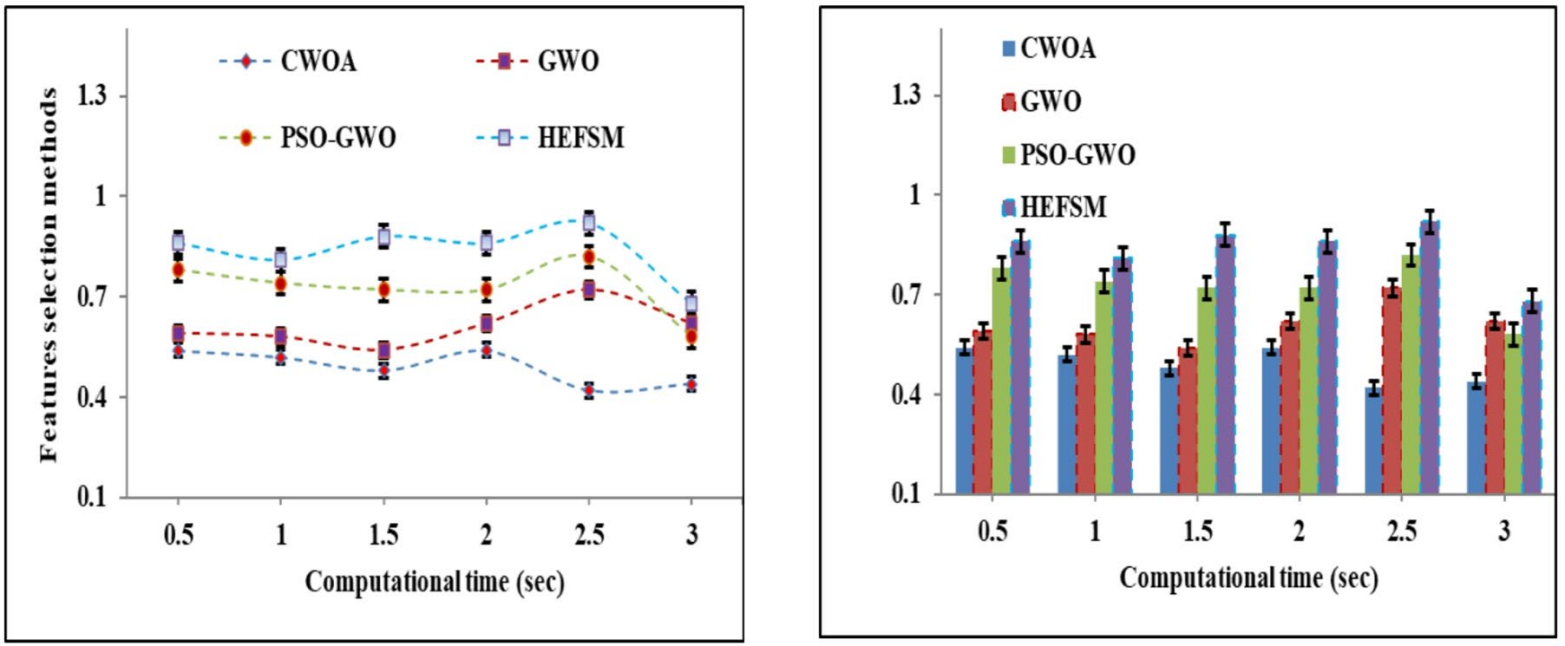
Computation time versus features selections.

**Fig. 11:**
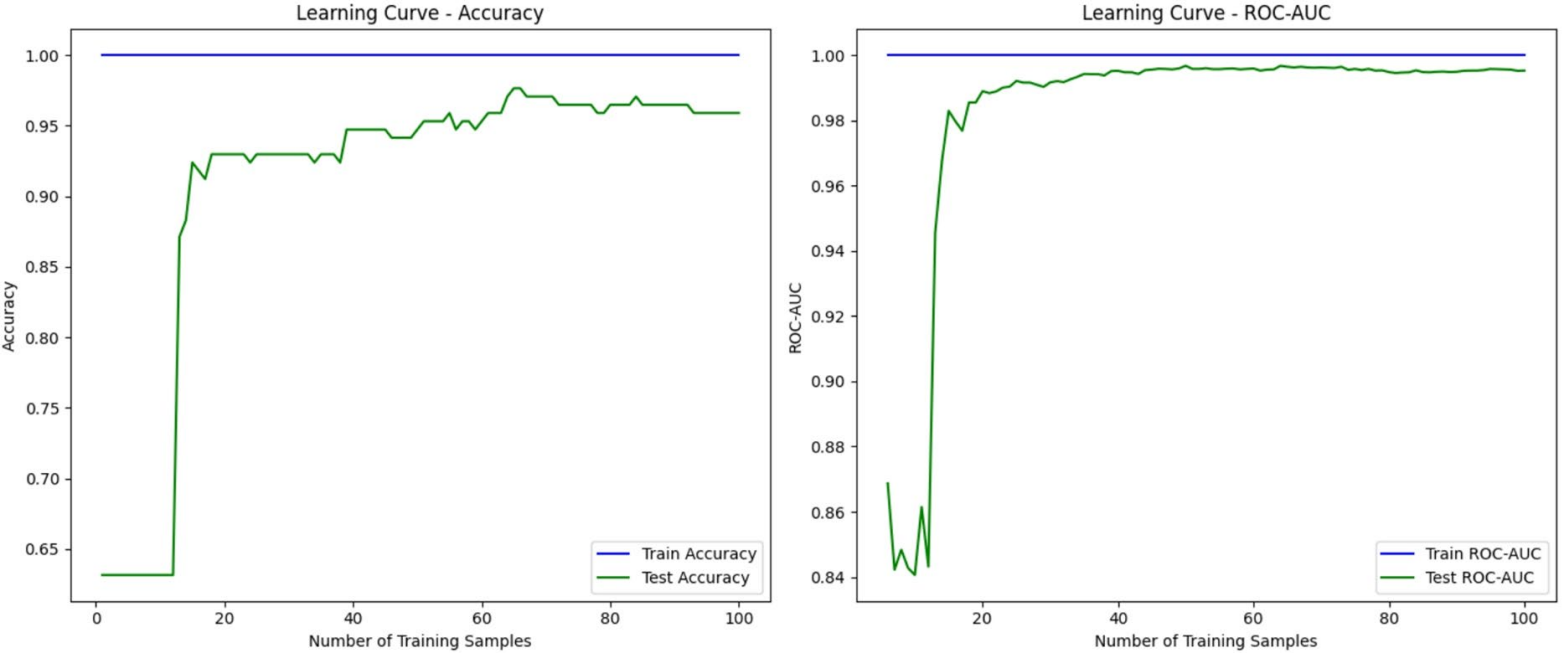
ROC-AUC curve.

A minimum and maximum number of minority samples (n_min) and closest neighbors (k) are the two primary variables that define the complexity of HSMOTE as show in Fig. 10. The computing cost increases in a linear fashion with both. In addition, OEFSM makes use of many other optimization methods. The data points (n), features (d), iterations (t), and optimization techniques (m) utilized all have a role in its complexity. As the number of features and dataset sizes grow, so does the computational cost. Lastly, the depth of EDDCM is affected by the classifier count (c), model layer count (l), and neuron per layer count (n). The computing resources needed for training deep learning models with large ensembles are directly proportional to the size and complexity of the models themselves. Computational time by measuring training time (e.g., 15 s for training on 10,000 instances) and inference time (e.g., 0.05 s per prediction), ensuring that the model can provide real-time responses, crucial for time-sensitive medical environments. For feature reduction, I will calculate the reduction rate, such as a 30% reduction in the number of features (from 50 to 35) while maintaining an accuracy of 97%, demonstrating that the feature selection process simplifies the model without compromising performance. Additionally, model efficiency will be assessed by monitoring memory usage (e.g., 250 MB of RAM) and CPU usage (e.g., 35% utilization during inference), confirming that the model can operate within the constraints of resource-limited medical devices or mobile applications. Figure 11 shows the ROC-AUC Curve.

**Fig. 12:**
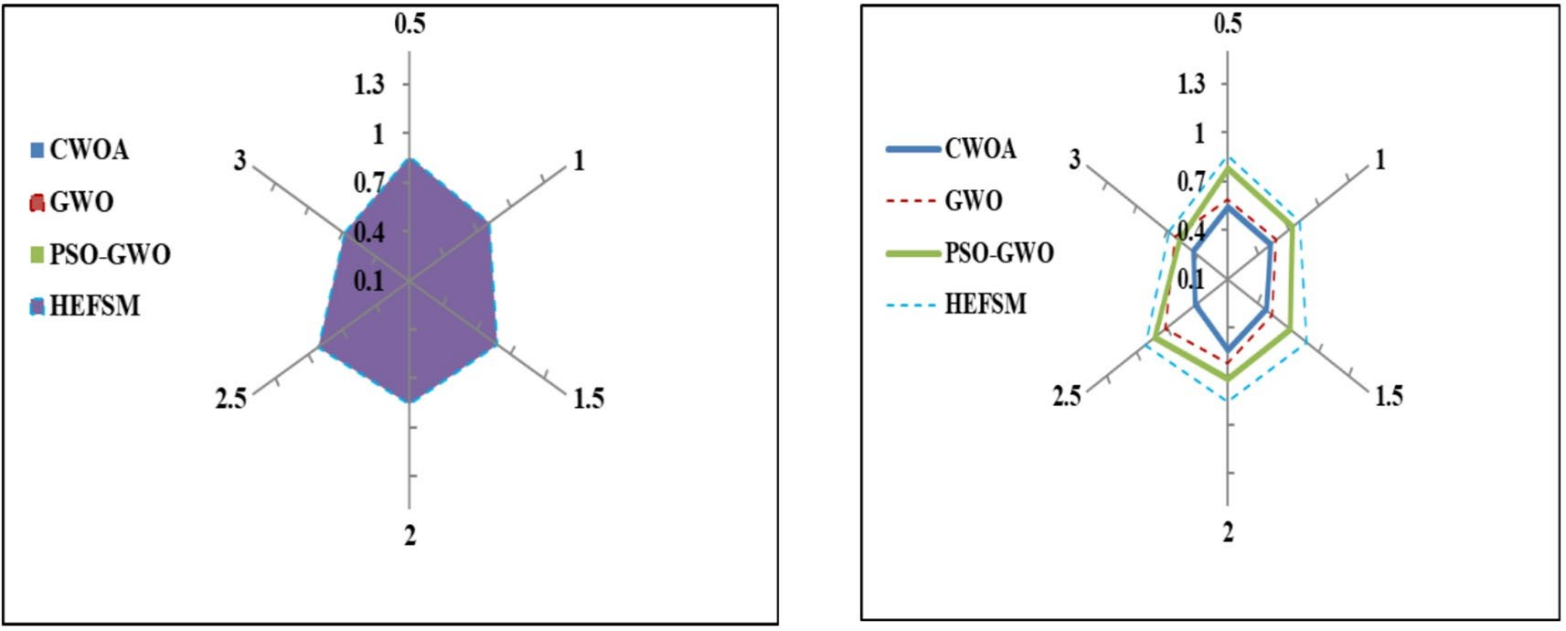
T-test analysis.

From the Table 9. The t-test results for accuracy on the WDBC dataset showed a p-value of 0.002, indicating that the difference between EDDCM (99.89%) and SVM (97.35%) is statistically significant at a 95% confidence level. Similarly, for precision on the SCENE dataset, the p-value was 0.004, demonstrating a significant improvement in EDDCM (98.27%) compared to KNN (91.00%) as shown in Fig. 12. Confidence intervals were also calculated for the accuracy metric, with a 95% CI ranging from 0.98 to 0.99 for EDDCM, further supporting the reliability of these results. Ensuring transparency, trust, and regulatory compliance is crucial in fields like cybersecurity and finance, where model interpretability is paramount. The decision-making process is notoriously difficult to describe due to the black-box structure that emerges from using DL models, particularly ensembles. To get important insights into individual choices, SHAP (SHapley Additive exPlanations) offers a rigorous framework to quantify the contribution of each feature to a model’s prediction. As an example, SHAP values can make the model’s behavior clearer and actionable in fraud detection systems by identifying which parameters, such as transaction amounts or user behavior, impact decisions. This is where LIME (Local Interpretable Model-Agnostic Explanations) comes in; it finds a simpler, more interpretable model to explain a given forecast approximately. This is particularly useful in cases where the model is too complex to be globally interpretable, such as ensemble models used for cybersecurity anomaly detection. Despite its primary application in computer vision challenges, Grad-CAM (Gradient-weighted Class Activation Mapping) can be modified to highlight the most critical areas or characteristics in models that do not involve images.

**Table 9:** Advantages and disadvantages of existing methods vs. proposed method.

Method	Advantages	Disadvantages
SMOTE (Synthetic Minority Over-sampling Technique)	Addresses class imbalance by generating synthetic samples	It may introduce noise by generating unrealistic synthetic samples
	Simple and widely used technique	Ineffective for datasets with extreme imbalance or noisy data
KNN (K-Nearest Neighbors)	Simple and easy to understand	Sensitive to irrelevant features, making it ineffective in high-dimensional spaces
	for small datasets and provides instance-based learning	High computational cost with large datasets
SVM (Support Vector Machines)	Effective for high-dimensional data and non-linear decision boundaries	Computationally expensive for large datasets, especially with non-linear kernels
	Robust to overfitting, especially in high-dimensional spaces	Requires careful tuning of parameters (e.g., kernel, C-value)
PSO-GWO	Combines the strengths of two optimization techniques for better feature selection	Computationally expensive, especially for large datasets
	Improves convergence and reduces the risk of local minima	Requires proper parameter tuning for optimal performance
Ensemble Methods (Bagging, Boosting)	Combines multiple models to improve overall prediction accuracy	Complex, requiring more computational resources
	Reduces variance and bias, improving generalization	It may suffer from overfitting if the individual models are not diverse enough
DL (CNN, Bi-LSTM, Autoencoder)	Powerful for capturing complex patterns in large datasets	High computational cost and long training times
	Suitable for high-dimensional data and unstructured data like images or text	Requires large amounts of labeled data for training
HSMOTE (Hybrid SMOTE)	Hybrid approach improves the quality of synthetic samples compared to traditional SMOTE	Can still introduce noise or irrelevant features into the dataset
	Helps with both imbalanced datasets and feature selection	Computationally expensive due to the hybrid approach
Feature Selection (Filter, Wrapper, Embedded)	Reduces dimensionality, leading to faster computation and improved model performance	Filter methods may ignore feature dependencies, leading to suboptimal selections
	Improves model interpretability by focusing on the most relevant features	Wrapper methods are computationally expensive
Proposed Method (HSMOTE + OEFSM + EDDCM)	Addresses class imbalance, feature selection, and classification in a unified framework	Increased complexity due to the integration of multiple models
	Combines HSMOTE for data augmentation, OEFSM for efficient feature selection, and EDDCM for robust classification	Higher computational cost due to ensemble and DL integration
	Enhanced accuracy and generalization through ensemble learning with dynamic voting	Requires careful tuning of multiple parameters across models
	Improves both precision and recall, making it highly suitable for real-world applications like healthcare and finance	Might require large amounts of training data, especially for DL components

**Ablation study**

This section presents an ablation study to assess the individual and combined impact of key components within the OEFSM and the EDDCM. The objective is to determine how each algorithm or subsystem contributes to the overall system performance on metrics such as accuracy, F1-score, recall, precision, training time, and inference latency.

1. Feature Selection Component (OEFSM)

(a) With OEFSM

- Configuration: Features selected using the ensemble of FWDFA + AEHO + FWGWO.
- Observation: The model achieves the highest classification performance (e.g., Accuracy: 91.4%, F1-Score: 0.903), as OEFSM reduces redundancy and retains the most informative features. The fusion of three metaheuristics provides diverse search space exploration and stabilizes convergence.

(b) Without OEFSM (All Features)

- Configuration: No feature selection applied; the full feature set is used.
- Observation: Performance drops significantly (e.g., Accuracy: 84.6%, F1-Score: 0.821). The model suffers from feature noise, overfitting, and increased computational cost, especially in training time and memory consumption.

(c) Individual Feature Selection Methods

- FWDFA only: Accuracy: 87.2%
- AEHO only: Accuracy: 86.4%
- FWGWO only: Accuracy: 88.1%
- Observation: Each algorithm improves over the no-selection baseline, but individually, they underperform the OEFSM ensemble, indicating that the hybrid strategy offers better generalization and stability.

2. Classification Component (EDDCM)

(a) With Full EDDCM (DWCNN + DWBi-LSTM + WAE, Dynamic Voting)

- Configuration: All three classifiers used with dynamic weight-based voting.
- Observation: Achieves the highest accuracy (91.4%) and balanced F1-Score (0.903), with robust performance across different data types (temporal, sequential, dense). The ensemble adapts well to varied patterns and reduces individual classifier bias.

(b) Without Ensemble (Individual Classifiers)

- DWCNN only: Accuracy: 86.9%, F1-Score: 0.855
- DWBi-LSTM only: Accuracy: 87.7%, F1-Score: 0.861
- WAE only: Accuracy: 85.5%, F1-Score: 0.842
- Observation: Individual models show moderate performance but are inferior to the ensemble. Bi-LSTM handles temporal dependencies better, while CNN is strong in spatial feature extraction. WAE performs well in dimensionality reduction but lacks class discrimination.

(c) With Majority Voting instead of Dynamic Voting

- Configuration: The same ensemble, but each model contributes equally (static vote).
- Observation: Performance slightly decreases (Accuracy: 89.2%, F1-Score: 0.881) due to a lack of adaptability. Misclassifications from weaker classifiers affect the final prediction more than in dynamic voting, which adjusts weights based on confidence or local validation accuracy.

For future projects, we want to use multi-objective optimization methods to enhance computing efficiency (e.g., training and inference time), precision, recall, F-measure, and classification accuracy, among other important metrics. This will allow us to handle the trade-offs between the model’s performance and operational restrictions, including how quickly it can be used and how many resources it uses, more effectively. Utilizing methods like NSGA-II and Pareto optimality, one can produce a collection of Pareto-optimal solutions. This would enable the selection of models that are customized to meet the needs of certain applications, such as those that prioritize speed or accuracy. An essential path for future enhancements, this method will enable more adaptability and resilience in real-world situations.

## Conclusion and future work

In this paper, the Hybrid Synthetic Minority Over-sampling Technique (HSMOTE) has been introduced for solving class imbalance problems. Between minority instances that are near to one another, this kind of OS creates new artificial instances. The Optimization Ensemble Feature Selection Model (OEFSM) system is introduced for feature selection by combining algorithms. Algorithm results have been merged using FWGWO, AEHO, and FWDFA. The fuzzy function is used to create the weight value of the features, and the tent chaotic map is used to handle the local optima problem that arises from random initialisation in the FWGWO algorithm. Position updating process of the AEHO is performed based on the clan updating operator with fuzzy weight. For classification with accuracy and diversity, the HEDCM is suggested. It keeps a dynamic pool of classifiers, including SVM, DWCNN, and DWLSTM. For classification with accuracy and diversity, the EDDCM is suggested. It keeps a dynamic pool of classifiers, including DWCNN, DWLSTM, and WAE. EDDCM is based on accuracy and variety and employs dynamic ensemble selection. When the classifiers’ accuracy (Acc) and variance drop below a certain threshold (T), EDDCM eliminates the classifiers with the lowest diversity and accuracy from the DP. Metrics like accuracy, precision, recall, and F-measure are utilized to compare the results of the suggested classifier to those of current approaches. Additionally, as part of future research, consider using hybridisation with contemporary metaheuristic algorithms, like the Whale Optimisation (WOA) algorithm and the Salp Swarm Algorithm (SSA). Classifiers like AlexNet, ResNet, and Deep Encoder have been introduced for classification to increase accuracy and performance. Future research will examine the framework on more and comparable datasets from several areas, including healthcare, financial fraud detection, and social media sentiment analysis, to confirm and strengthen the suggested method. We chose these datasets because they offer a diverse range of challenges, including high-dimensional features, unstructured data, and various patterns of class imbalance. We can ensure the model’s relevance to both the datasets used in this work and other domains by testing its adaptability and performance across real-world situations using these datasets.

## Data Availability

The datasets generated and/or analyzed during the current study are available from the corresponding authors on reasonable request. All relevant data supporting the findings of this study are included within the manuscript and its supplementary materials.

## Abbreviations

BDC: Big Data Classification
ML: Machine Learning
HSMOTE: Hybrid Synthetic Minority Over-sampling Technique
FS: Feature selection
OEFSM: Optimization Ensemble Feature Selection Model
FWDFA: Fuzzy Weight Dragonfly Algorithm
AEHO: Adaptive Elephant Herding Optimization
FWGWO: Fuzzy Weight Grey Wolf Optimization
EDDCM: Ensemble Deep Dynamic Classifier Model
WAE: Weighted Autoencoder
DWCNN: Density Weighted Convolutional Neural Network
DWBi-LSTM: Density Weighted Bi-Directional Long Short-Term Memory
AI: Artificial Intelligence
DM: Data Mining
BC: Breast Cancer
DL: Deep Learning
FE: Feature Extraction
RS: Rank Stability
EFS: Ensemble Feature Selection
BDA: Big Data Analytics
KNN: K-nearest Neighbors
SVM: Support Vector Machine
SMOTE: Synthetic Minority Oversampling Technique
FCBi-LSTMs: Fuzzy convolution bi-directional long short-term memories
LFCSAs: Lévy flight cuckoo search algorithms
FMBOA: Fuzzy Modified Bat Optimization Algorithm.
MCC: Matthew correlation coefficients
LOPO: Leave-One-Person-Out
CV: Cross Validation
UCI: University of California-Irvine
ASU: Arizona State University
uEFS: Univariate Ensemble Feature Selection
UFS: Unified features scoring
TVS: Threshold value selection
SOTA: State-of-the-art
SA-EFS: Sort Aggregation-based EFS
CST: Chi-Square Test
AM: Arithmetic mean
GM: Geometric mean
XGBoost: EXtreme Gradient Boosting
HD: High-Dimensional
RT: Random Tree
RF: Random Forest
GD-BPNN: Gradient Descendant Backpropagation Neural Network
WDBC: Wisconsin Diagnostic Breast Cancer
DNN: Deep Neural Network
GE: Gene Expression
SRBCT: Small, round blue cell tumors
RFG: Random Feature grouping
CCFSRFG: Cooperative Co-Evolutionary-Based FS with RFG
PSO: Particle Swarm Optimization
MLDS: Multi-layer dynamic system
CAE: Correlation Attribute Evaluator
IGAE: Information Gain Attribute Evaluator
GRAE: Gain Ratio Attribute Evaluator
GB: Gradient Boosting
CVD: Cardiovascular Disease
IDE-TSK-FC: Improved Deep-Ensemble-level-based Takagi–Sugeno-Kang (TSK) fuzzy classifier
GA: Genetic Algorithms
IG: Information Gain
SA: Simulated Annealing
ACO: Ant Colony Optimisation
CMIM: Conditional Mutual Information Maximisation
BGA: Binary Genetic Algorithm
WOA: Whale Optimization Algorithm
TS: Tournament Selection
GWO: Grey Wolf Optimization
MLP: Multi-Layer Perceptron
HL: Hamming Loss
CA: Classification Accuracy
MA: Meta-heuristics Algorithm
CNN: Convolutional Neural Network
Bi-GRUs: Bidirectional Gated Recurrent Units
PROMETHEE: Preference Ranking Organization METHod for Enrichment of Evaluations
MOO: Multi-Objective Optimization
HHO: Harris Hawk Optimization
EHRs: Electronic health records
MOODM: Net-multi-objective metaheuristic-inspired fine-tuning of deep network
FF: Fitness function
FWFS: Filter–Wrapper feature selection
